# A tissue-specific profile of miRNAs and their targets related to paeoniaflorin and monoterpenoids biosynthesis in *Paeonia lactiflora* Pall. by transcriptome, small RNAs and degradome sequencing

**DOI:** 10.1371/journal.pone.0279992

**Published:** 2023-01-26

**Authors:** Pan Xu, Quanqing Li, Weiqing Liang, Yijuan Hu, Rubing Chen, Kelang Lou, Lianghui Zhan, Xiaojun Wu, Jinbao Pu

**Affiliations:** 1 Center for Medicinal Resources Research, Zhejiang Academy of Traditional Chinese Medicine, Hangzhou, Zhejiang Province, China; 2 Key Laboratory of Research and Development of Chinese Medicine of Zhejiang Province, Hangzhou, Zhejiang Province, China; 3 Department of Pharmacy, Zhejiang Xiaoshan Hospital, Hangzhou, Zhejiang Province, China; Texas A&M University System, UNITED STATES

## Abstract

*Paeonia lactiflora* Pall. (*Paeonia*) has aroused many concerns due to its extensive medicinal value, in which monoterpene glucoside paeoniflorin and its derivatives are the active chemical components. However, little is known in the molecular mechanism of monoterpenoids biosynthesis, and the regulation network between small RNAs and mRNAs in monoterpenoids biosynthesis has not been investigated yet. Herein, we attempted to reveal the tissue-specific regulation network of miRNAs and their targets related to paeoniaflorin and monoterpenoids biosynthesis in *Paeonia* by combining mRNA and miRNA expression data with degradome analysis. In all, 289 miRNAs and 30177 unigenes were identified, of which nine miRNAs from seven miRNA families including miR396, miR393, miR835, miR1144, miR3638, miR5794 and miR9555 were verified as monoterpenoids biosynthesis-related miRNAs by degradome sequencing. Moreover, the co-expression network analysis showed that four monoterpenoids-regulating TFs, namely *AP2*, *MYBC1*, *SPL12* and *TCP2*, were putatively regulated by five miRNAs including miR172, miR828, miR858, miR156 and miR319, respectively. The present study will improve our knowledge of the molecular mechanisms of the paeoniaflorin and monoterpenoids biosynthesis mediated by miRNA to a new level, and provide a valuable resource for further study on *Paeonia*.

## Introduction

*Baishao*, the dry bark-free root of cultivated *Paeonia lactiflora* Pall. (*Paeonia* in short), has been employed to treat rheumatoid arthritis, dysmenorrhea, hepatitis, muscle cramps and spasms, depression, diabetes and fever for thousands of years in East Asia [1, 2]. Since paeoniflorin was first discovered in *Paeonia* in 1963, a series of glucosides have been found, including paeoniflorin, oxypaeoniflorin, albiflorin, benzoylpaeoniflorin, lactiflorin, paeonin, oxybenzoylpaeoiniflorin, paeoniflorigenone, galloylpaeoniflorin, and paeonol [3–5], most of which are monoterpene glucosides. As important chemical components in *Paeonia*, monoterpene glucosides usually have one pinane skeleton and one acetal structure, which belong to dicyclic monoterpene glucosides. Paeoniflorin is the most abundant among them, reaching more than 90% [6] and accounts for various pharmacological effects of *Paeonia*. It has been highlighted by researchers due to its diverse properties, such as anti-inflammatory, analgesic, hepatoprotective, antioxidant, anticancer, hypoglycemic effects, and it protects cardiovascular and cerebrovascular systems, nervous systems and other activities [7–10].

The biosynthesis of paeoniflorin and its derivatives is controlled by the terpenoid pathways, which are usually divided into three stages, including the stage of isoprene pyrophosphate (GPP) formation as the precursor, the stage of skeleton formation and the stage of post-modification. The synthesis of GPP is mainly realized via mevalonate (MVA) and 1-deoxyd-dxylose-5-phosphate/methylerythritol-4-phosphate (DXP/MEP) pathways [11]. Most of the enzymes that participated in the terpenoid pathway have been identified and characterized along decades of research, including hydroxymethylglutaryl-CoA synthase (HMGS), 3-hydroxy-3-methylglutaryl-CoA reductase (HMGR), mevalonate kinase (MVK), phosphomevalonate kinase (PMK) in MVA pathway [12], 1-deoxy-D-xylulose-5-phosphate synthase (DXS), 1-deoxy-D-xylulose 5-phosphate reductoisomerase (DXR), 2-C-mehyl-D-erythritol 4-phosphate cytidyltransferase (IspD), 4-diphosphocytidyl-2-C-methyl-D-erythritol kinase (IspE), 2-C-mehyl-D-erythritol 2,4-cyclodiphoaphate synthase (IspF), 1-hydroxy-2-methyl-2-(*E*)-butenyl 4-diphosphate synthase (HDS), 1-hydroxy-2-methyl-2-(*E*)-butenyl-4-diphosphatereductase (HDR), isopentenyl diphosphate isomerase (IDI) in DXP/MEP pathway [13, 14]. and monoterpene synthase (Mono-TPS) [15], cytochrome P450 (CYP450s) [16] and glycosyltransferase (UGTs) [17] in the last two stages. There also have been extensive studies on the genetic regulation of terpenoid biosynthesis, which have highlighted the significant regulatory role of various transcription factor families, such as APETALA2 (AP2) / ethylene response factors (ERF), basic leucine zipper (bZIP), basic helix-Loop-helix (bHLH), MYB, WRKY, and so on [18–22]. Moreover, they could be regulated by functional gene clusters [23, 24].

Besides these typical pathways, recent studies have found that miRNAs exert a crucial impact on regulating the biosynthesis of plant natural products [25, 26]. MiRNAs are a class of single-strand, small, non-coding RNAs, consisting of 21–24 nucleotides that negatively regulate gene expressions at the post-transcriptional level [27, 28]. Furthermore, they are vital for the plant developmental process, cell differentiation, morphogenesis and response to various abiotic and biological stresses via interactions with their specific targeted mRNAs [29–31]. For instance, miR5072 was identified to cleave acetyl-CoA C-acetyltransferase (ACAT) and related to the biosynthesis of terpenoids and tanshinones in *Salvia miltiorrhiza* [32]. With the development of sequencing techniques, several researchers have utilized small RNA sequencing to profile miRNAs in plants. For instance, miR5254, miR3897-3p, miR165a-3p and miR6450a in *P*. *lactiflora* were response to *Botrytis cinerea* infection [33]. The etilation of the flower of *P*. *lactiflora* was regulated by miR156e-3p targeting squamosa promoter binding protein-like gene (SPL1) [34]. These studies have indicated the role of small RNAs in paeoniflorin and monoterpenoids biosynthesis.

To thoroughly reveal the impact of miRNAs on the paeoniflorin biosynthesis, it`s fundamental to accurately identify their targeted genes and explore their interactions. The major approaches for identifying miRNAs target genes were computational target prediction, 5`RACE and degradome sequencing [35, 36]. Among them, degradation sequencing is considered to be a powerful method for connecting high-throughput sequencing and screening miRNA targets in large scales [37]. Combining the advances of high-throughput sequencing technique and bioinformatics analysis, it has been extensively applied to identify miRNA target genes in *Arabidopsis*, rice and many other plants due to its high efficiency and accuracy.

In this study, we focus on investigating the small RNA-mediated paeoniflorin and monoterpenoids biosynthesis in *Paeonia*. Four tissues of root, leaf, flower and fruit, which displayed different paeoniflorin accumulation, were sampled. The facts that paeoniflorin existed in each tissue but the content varied greatly indicating that paeoniflorin is synthesized in all four tissues of *Paeonia* and the content differences of paeoniflorin in each tissue may be caused by the expression difference of these biosynthesis-related genes. The tissue-specific differentially expressed pattern facilitated us to investigate the molecule-related foundation of paeoniflorin biosynthesis and transport. Thus, a transcriptome-wide study on different tissues of *Paeonia* was carried out by combining RNA, sRNA and degradome sequencing. As far as we know, this is the first report on how miRNAs regulate paeoniflorin biosynthesis. Identification of the tissue-specific miRNAs and their target genes will enhance our comprehending on their role in a variety of metabolic pathways, and provide new insights into the regulatory network of paeoniflorin and monoterpenoids biosynthesis.

## Materials and methods

### Plant materials collection

**Fig 1:**
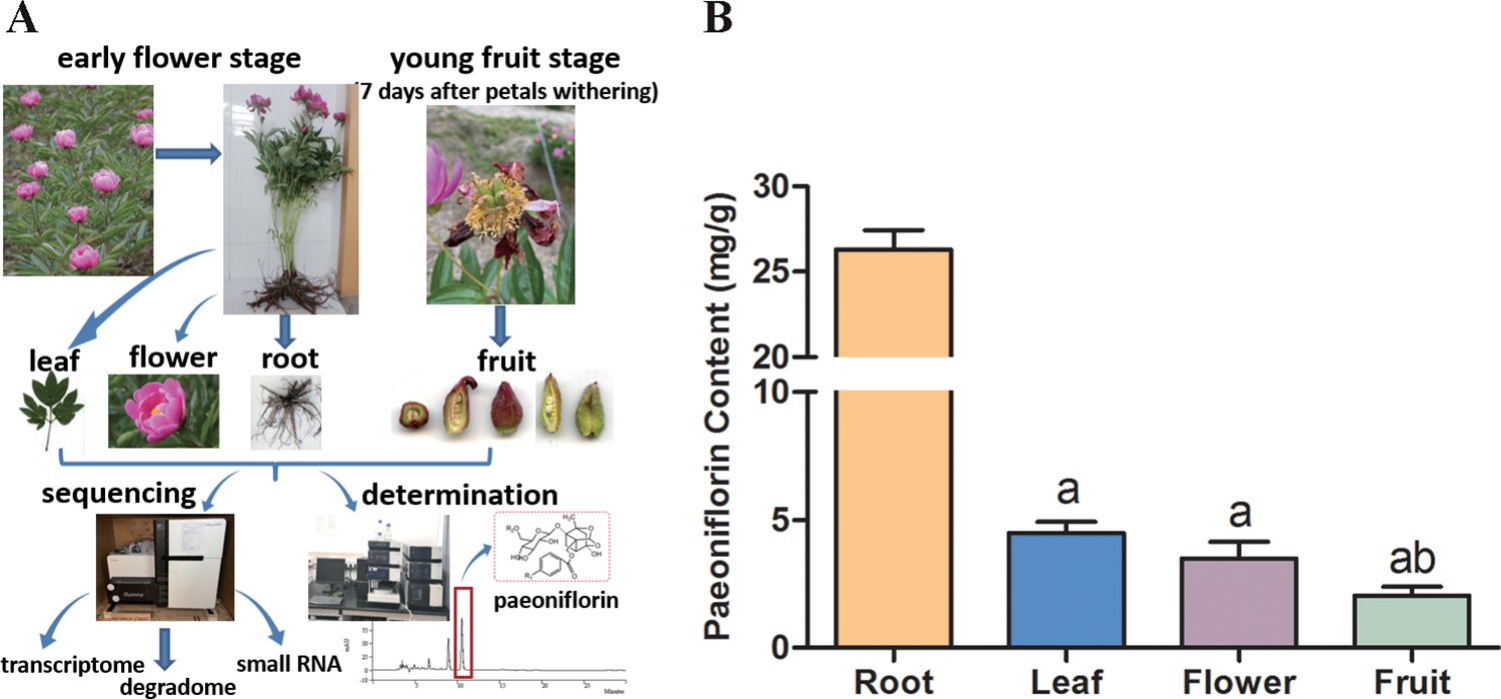
Quantitative analysis of paeoniflorin in different tissues of *Paeonia*. (A Sampling process and four different tissues of *Paeonia*, B The contents of paeoniflorin in four different tissues, a represents significant differences at p<0.05 by comparing root, b represents significant differences at p<0.05 by comparing leaf).

*Paeonia* was cultivated in the experiment field of Zhejiang Academy of Traditional Chinese Medicine, China. The newly flowering *Paeonia* plants were selected for harvesting at the early flower stage, and the samples of roots, leaves and flowers were acquired respectively. In addition, the follicles of *Paeonia* were collected 7 days after the petals withering, which at the young fruit stage. Samples were collected from plants at the same phenological period to eliminate the influence of different growth stages on the same kind of tissue as much as possible. Approximately 3 g of tissues were taken from each plant, quickly frozen with liquid nitrogen and reserved at -80°C for further analysis. Each sample was pooled from individual plants, and two biological replicates were collected. Part of collected tissues were used for paeoniflorin content determination, while the other part of collected tissues was used for RNA extraction. Total RNA was extracted and purified by TRIzol reagent (Invitrogen, Carlsbad, CA, USA) according to the manufacturer’s instructions. The amount and purity of total RNA were analyzed by Bioanalyzer 2100 (Agilent, CA, USA) and RNA 6000 Nano LabChip Kit (Agilent, CA, USA) with RIN value being at least 7.0. The purified RNA was used for transcriptome, small RNA and degradome sequencing. The sampling and analysis process were showed in Fig 1A.

### Quantitative analysis of paeoniflorin in different tissues of *Paeonia*

The contents of paeoniflorin in four different tissues (leaf, root, flower and fruit) of *Paeonia* were quantitatively determined by HPLC. In short, the low-temperature dried tissues were ground to a uniform powder for subsequent testing. First, 0.1g samples were ultrasonic-extracted with 35 ml 50% ethanol solution for 30 min at the condition of room temperature, 240W of power and 45 kHz of frequency. After 0.22 μm membrane filtration, 2 ml extract solution was prepared for HPLC detection.

The HPLC analysis of the samples and paeoniflorin (standard) was performed on a Varian Prostar HPLC system (Varian, USA, CA) with a YMC-Pack-ODS-A C_18_ analytical column (4.6mm×250mm, 5μm, YMC Co., Ltd., Japan, Kyoto). The mobile phase was employing acetomitrile-0.1% phosphorus acid solution (14:86) with an isocratic elution. The flow rate was set as 1.0 ml/min and column temperature was kept at 30°C. The samples were detected on the wavelength of 230nm. According to the retention time, these components were determined and compared with paeoniflorin standard. The content of paeoniflorin in samples was calculated by conventionally comparing with the peak area of the standard. Meanwhile, the samples were dried at 105°C until constant weight to measure their moisture contents. And the moisture content was deducted when the paeoniflorin content was finally calculated. The paeoniflorin contents of all tissues were expressed as mean value and standard deviation (SD) value. One-way ANOVA was used for the comparison among the groups, and Tukey`s Multiple Comparison Test was used for pairwise comparison among each group. P value less than 0.05 indicated a significant difference between two groups.

### Transcriptome sequencing and de novo assembly analysis

For RNA library construction, poly-T oligo-attached magnetic beads were used to purify the RNA from total RNA (approximately 5μg) with twice purification. Next, the mRNA was fragmented into small pieces via divalent cations at high temperature. Then the final cDNA library was established by reverse-transcribed of the cleavage RNA pieces in accordance with the guidance for the mRNASeq Sample Preparation Kit RS-122-2103 (Illumina, San Diego, USA). The average insert size for the libraries was 300 bp (±50 bp). The prepared libraries were then sequenced on an IlluminaHiseq 2000 (LC Sciences, San Diego, USA) platform according to the vendor’s recommended protocol, and 150bp paired-end reads were generated. After filtering the primers, adapters and reads with low-quality and >5% bases using Cutadapt software [38], de novo assembling of the transcriptome was performed by Trinity 2.4.0 [39] with default parameters on the foundation of an overall 60.61GB RNA-seq data. Benchmarking Universal Single-Copy Orthologs (BUSCO) [40] were used to assess transcriptome assembly and annotation completeness. Unigenes were then queried against the Swiss-Prot, Non-redundant (Nr), Protein family (Pfam), Kyoto Encyclopedia of Genes and Genomes (KEGG), eukaryotic Orthologous Groups (KOG), Gene Ontology (GO) public databases by BLASTx with an E-value <10^−5^ to obtain annotation. RPKM (Reads Per Kilobase per Million mapped reads) were employed to evaluate genetic expressing levels. Differential expression analysis were performed using edgeR [41] to obtain p value, and Benjamini-Hochberg (BH) algorithm was used to adjust p value for controlling false discovery rate (FDR). To define differentially expressed genes, we used fold change (FC) ≥1.5 and FDR ≤0.05 as cutoff. Subsequently, GO and KEGG enrichment analyses and the heatmaps of differential expressed genes (DEGs) among four tissues were performed using the OmicStudio tools at https://www.omicstudio.cn/tool.

GeneMANIA app in cytoscape version 3.7 was employed to discover genes modulated by identified transcription factors (TFs) [42]. Genes screened and annotated as monoterpenoid biosynthesis related genes (TPs) along with TFs activities were adopted for the purpose of drawing a co-expressing network. *Arabidopsis* co-expression network was employed as a reference to query [43], genes with the greatest normalized weight (high associated genes) were forecasted to be related genes correlated with a specific role. The normalized weight was calculated by default weighting method of GeneMANIA app.

### sRNA sequence and miRNA identification

For sRNA library construction, small RNA library was prepared from total RNA (approximately 1μg) according to protocol of TruSeq Small RNA Sample Prep Kits (Illumina, San Diego, USA). Afterwards, single-end sequencing (36bp) was performed on an Illumina Hiseq2500 at the LC-BIO (Hangzhou, China) following the manufacturer’s procedure. Data processing followed the procedures as described in a previous research [32] to identified the conserved and novel miRNAs in *Paeonia*. The novel miRNAs were screened according to the latest published criteria of novel miRNA [44]. Only non-coding sequences that could form a stem-loop duplex with <4 mismatched bases and derived from opposite stem-arms were considered as a potential novel miRNA candidate.

### Degradome sequencing, target identification and analysis

The RNA of all eight different samples from four tissues was mixed equally for degradome library construction. About 20 μg of total RNA were used for the sequencing. Pipeline v1.5 software of Illumina was adopted to obtain the raw sequencing reads by removing adaptor sequences and low quality sequence. As previously described, the extracted sequence with a length of 20nt and 21nt were then employed to identify potential cleavage targets by CleaveLand pipeline [45]. MiRU [46] algorithm with default parameters was used for target prediction. Next, the cleavage sites were detected according to the criteria previously proposed [47]. Only the transcripts with distinct cleavage signals were consider to be regulated by specific miRNAs. For easily analyze the miRNA and RNA degradation patterns, the target was then categorized according to the T-plots, which generated by the distribution of signature and abundance of transcripts [48]. Commonly, the identified cleavage sites were group into five different categories (0–4). The target fall into Category 0 were the most significant with only one maximum value in target plot. Category 1 comprised the most abundant fragements with more than one maximum value in target plot. Category 2 fragements were present at abundances between the median and the maximum. Category 3 comprised the fragements with abundances below or equal to the median. Category 4 sequence were only one raw read at the degradation position. The smaller the category classification number, the more reliable the result is.

### qRT-PCR verification of miRNAs and targeted genes

A total of 12 differential expressed miRNA and 11 targeted genes were selected to verify the interaction between miRNA and targets by qRT-PCR. For miRNA, forward primers were developed according to the sequence of miRNA itself (S1 Table), and universal primers provided by miRcute Plus miRNA First-Strand cDNA Kit (Tiangen, Beijing, China) were employed as reverse primers. U6 snRNA was adopted as an inner reference. The qRT-PCR were performed via miRcute Plus miRNA qPCR Kit (SYBR Green) (Tiangen, Beijing, China). The primers for target genes were designed using primer 5.0 (Primer-E Ltd., Plymouth, UK) (S2 Table). Plactin [6, 49] (GenBank: JN105299; Available online: http://www.ncbi.nlm.nih.gov) was utilized as an internal control. Reverse transcription reactions and RT-qPCR reactions were completed with the FastKing RT Kit (with gDNase) (Tiangen, Beijing, China) and SuperReal PreMix Plus (SYBR Green) Kit (Tiangen, Beijing, China). All reactions were performed for three times for each sample. The comparative expressing level of miRNA and mRNA was calculated according to the 2^-ΔCt^ approach [50].

### Target genes annotation and co-expressing network establishment

GO and KEGG enrichment analysis were carried out to determine the function of targets. Subsequently, the interaction relationship of mRNA and miRNA which obtained by degradome analysis was used for the construction of whole miRNA-mRNA interaction network and was visualized in Cytoscape.

## Results

### Quantitative analysis of paeoniflorin in different tissues of *Paeonia*

The content of paeoniflorin in four different tissues of *Paeonia* was determined (Fig 1B). The results demonstrated that the content of paeoniflorin in the root was the highest, reaching 26.31mg/g, which was significantly higher than that in the other three tissues (P<0.05). Among the other three tissues, the content of paeoniflorin in the fruit was the lowest (2.04mg/g), which showed a significant difference (P<0.05) compare to that in the leaf. The content of paeoniflorin in the flower was slightly higher than that in the fruit (3.50mg/g), whereas the content of paeoniflorin in the leaf reached the highest level of 4.48mg/g. There was no significant difference (P>0.05) between leaf and flower. The above results indicated that the content of paeoniflorin exhibited obvious tissue differences in root, leaf, flower and fruit. These results also indicated the rationality of taking root as the medicinal part in the clinic.

### Tissue-specifically expressed transcripts in *Paeonia*

**Fig 2:**
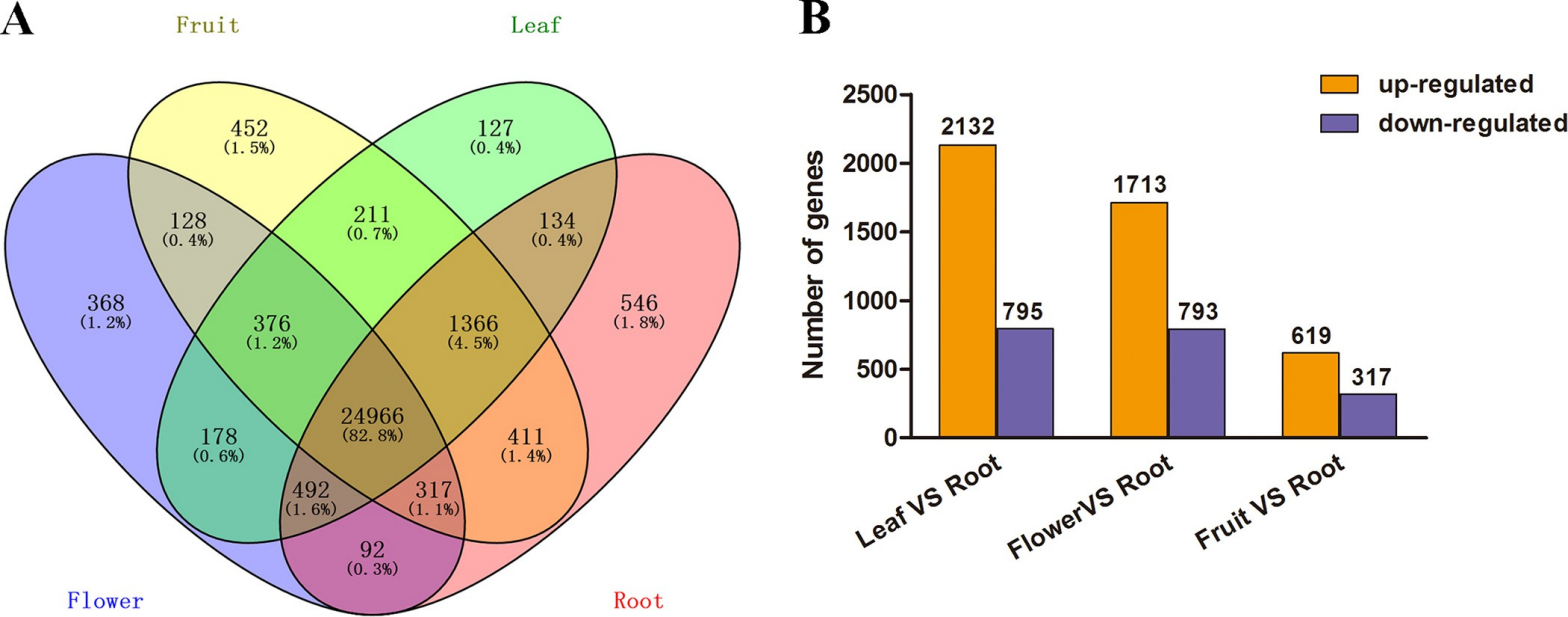
Tissue-specifically expressed genes in *Paeonia*. (A The spatial expressing patterns of genes in four different tissues, B Number of differentially expressed genes between different tissues).

To identify monoterpenoids-biosynthesis-related genes and regulation network, transcriptomes of eight samples from four different tissues of *Paeonia* were sequenced (Table 1). An overall 418538930 raw reads were obtained from the RNA-seq library by Illumina HiSeqTM 2000 sequencing. A total of 411442976 clean reads were obtained after removing the primers, adapters and low-quality reads. Among them, 39758080 (91.42%) and 30399492 (94.00%) reads in flowers, 44392652 (83.38%) and 40901054 (85.52%) reads in roots, 49173430 (91.85%) and 42795795 (90.51%) reads in leaves, and 49421130 (89.07%) and 39919793 (86.96%) reads in fruits, were mapped to the reference genome, respectively. The entire clean reads from the eight RNA-seq data sets were combined for transcript assembling. As a result, 41503 transcripts that could be assigned to 30177 unique genes (unigenes) were assembled (S1 File). The length of transcripts was range from 201 nt to 7928 nt, with the average length of 1308 and 43.39% average GC content. We also checked the completeness of de novo assembled unigenes based on BUSCO assessment. The results (S1 Fig) showed that 81.46% transcripts were complete transcripts and 5.41% were missing, indicating a high quality of *Paeonia* genome assembly. The transcriptome-wide difference amongst the four tissues of *Paeonia* was investigated on the foundation of the RNA-seq data. Besides 24966 (82.8%) transcripts expressed in all tissues, 546 (1.8%) transcripts were only expressed in root, and 452 (1.5%) were only expressed in fruit, while 368 (1.2%) were only expressed only in flower, and 127 (0.4%) were only expressed in leaf, and most of them exhibited a low expressing level. Furthermore, the remaining transcripts were expressed in two or three different tissues (Fig 2A). In addition to using RPKM-based algorithm to calculate the gene expression level of assembling transcription in every tissue, an expressing-level-related contrast was performed for every two-tissue combination (take the root as a reference). As a result, there were 2927 DEGs identified between leaf and root, 2506 DEGs between flower and root, and 935 DEGs between fruit and root (S2 File). Among them, there were 2132, 1713 and 619 up-regulated genes, while 795, 793 and 317 genes exhibited down regulation, separately (Fig 2B).

**Table 1:** Summarization of transcriptome and small RNA sequence data.

Tissue	ID	Transcriptome sequence			Small RNA sequence		
		Clean reads	read used rate(%)	Mapping rate (%)	Clean reads	MiRNA	Known miRNA
Leaf	Leaf-1	53534304	98.39	91.85	5207890	173	153
	Leaf-2	47280526	98.49	90.51	7096767	160	141
Root	Root-1	53240730	97.94	83.38	5736916	157	143
	Root-2	47823836	98.31	85.52	5302298	146	132
Flower	Flower-1	43488630	98.36	91.42	11097750	196	178
	Flower-2	64682204	98.16	94.00	11339971	178	160
Fruit	Fruit-1	55486088	98.49	89.07	5770059	145	126
	Fruit-2	45906658	98.36	86.96	8525420	150	131
Total		411442976	/	/	60077071	279	257

**Fig 3:**
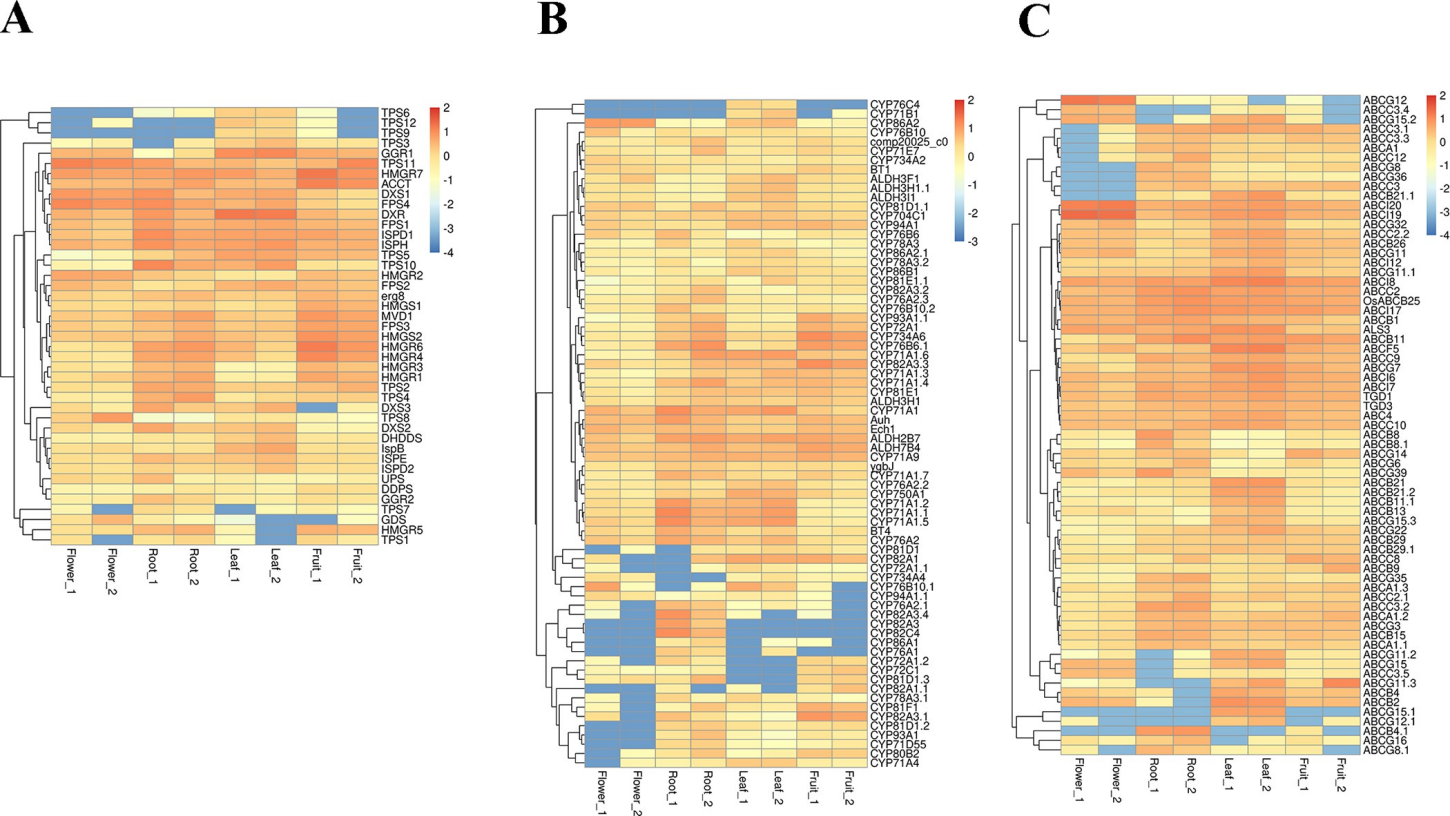
Hierarchical cluster analysis of DEGs involving in the monoterpenoids biosynthesis pathways in different tissues of *Paeonia*. (A The DEGs in terpenoid backbone and monoterpenoids biosynthesis pathway, B The DEGs in limonene and pinene degradation pathway, C The DEGs in transport pathway).

To further investigate the biological functions of the DEGs, the GO and KEGG annotations of the DEGs were screened and the results were listed in the S2 and S3 Figs and S3 File. Here, we focus on the functional categories involved in biosynthesis and transportation of secondary metabolites, like “Metabolic process”, “Secondary Metabolite Biosynthesis”, “Catalytic activity” and “Transport” which appeared in the two selected annotation and classification databases. The key enzymes encoding the genes of whole monoterpenoids biosynthesis pathways, which ranged from the glycolysis to terpenoid backbone to monoterpenoids, were the most pivotal ones. Overall, we identified three significantly enriched KEGG pathways and eight significantly enriched GO enrichment biological processes related to the monoterpenoids biosynthesis, such as terpenoid backbone biosynthesis, geranylgeranyl-diphosphate geranylgeranyl-transferase activity, monoterpenoid biosynthesis, isoprenoid biosynthetic process and limonene and pinene degradation, etc. There were 115 and 69 DEGs annotated to the monoterpenoids biosynthesis pathways and transport processes respectively, and the hierarchical cluster analysis of DEGs involved in these pathways was presented in Fig 3. The expression levels of *HMGR1*, *HMGS3*, *HMGR5*, *DXS2*, *DXS3*, *IspG*, *IspH*, *IspD1*, *FPS1*, *TPS10*, *TPS2*, *TPS4*, *CYP71A1*, *CYP82A3*, *CYP82A4* were the highest in root compared with other tissues. This is also compliance with the previous results of high expression and accumulation of paeoniflorin in roots.

### Co-expression network construction between monoterpenoids biosynthesis-related gene and transcription factors

**Fig 4:**
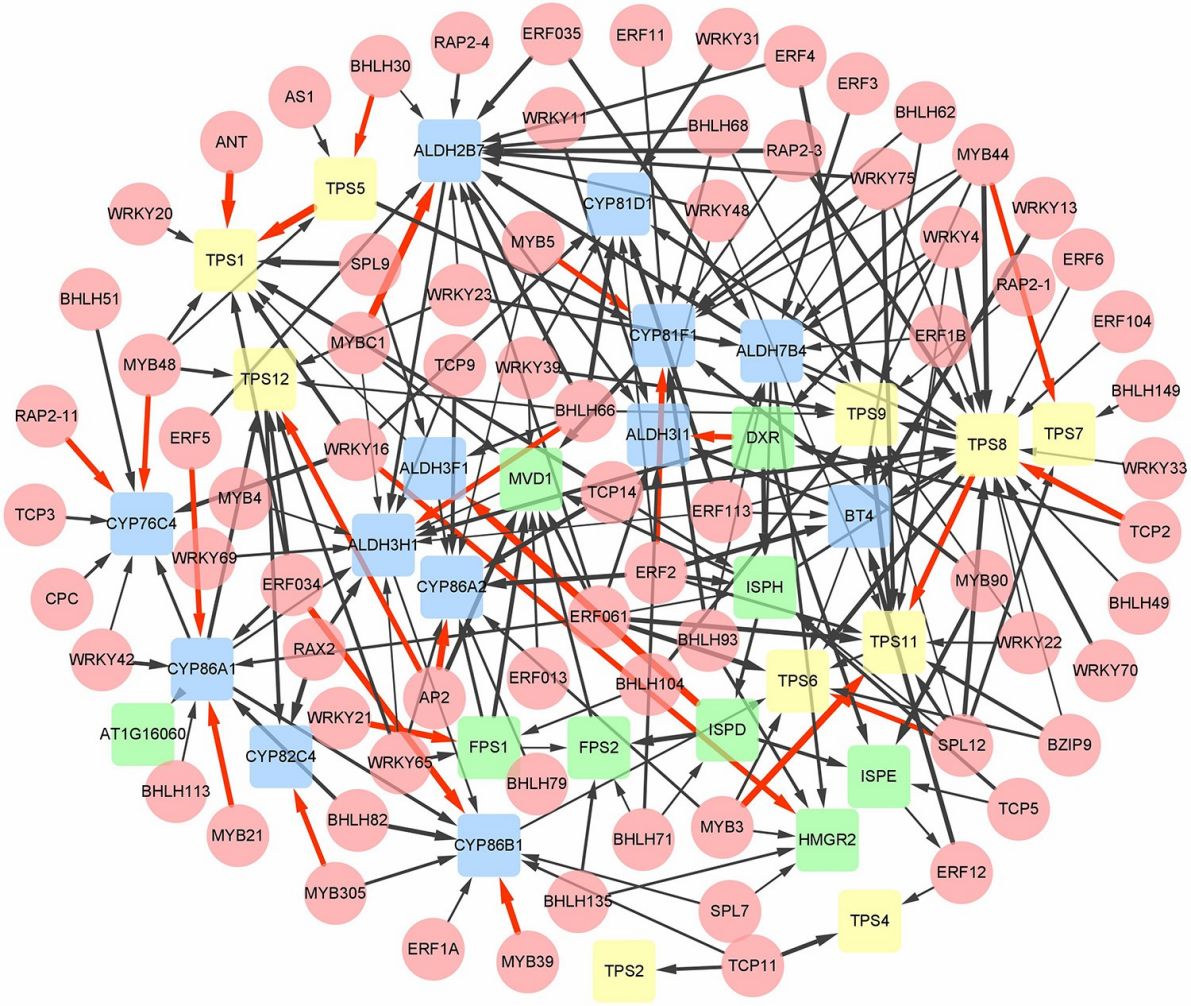
Genetic regulation network between transcriptional factors (TFs: Round nodes) and monoterpenoids biosynthesis genes (TPs: Round rectangle nodes). (Edges denote the association between nodes and the width of every edge denotes normalized co-expressing weight. Red edges represent high association between screened TFs and TPs. green round rectangles represent terpenoid backbone biosynthesis genes, yellow round rectangles represent monoterpenoids synthase genes and blue round rectangles represent the genes associated with post-modification stage of monoterpenoids biosynthesis).

Extensive studies on the genetic regulation of terpenoid biosynthesis have shown that various TFs play significant regulatory roles. A total of 161 DEGs were screened out and annotated as TFs. To reveal the association between identified TFs and monoterpenoid biosynthesis genes (TPs), the GeneMANIA App in Cytoscape software was employed for network analysis. According to guilt-by-association, a co-expressing network assay could be employed to predict gene function with high confidence. Two related genes in the co-expression network are often involved in the identical or associated functions [43, 51]. To predict the regulatory effects of identified transcription factors on monoterpenoid biosynthesis genes, *Arabidopsis* co-expression network was employed as a reference to query, and eventually genes with the greatest normalized weight were abstracted and explored. The results revealed that a total of 75 TFs and 32 TPs were involved in the co-expression network. *MYB*, *BHLH*, *WRKY* and *AP2/ERF* were the main TF families to regulate TPs. According to the normalized weight, the top 30 co-expression relationships ranging from 0.002117 to 0.001143 are listed in the S3 Table. The resultant co-expression network showed that *TPS* genes were highly co-expressed, indicating that their functions were highly similar, which also increased the credibility of KEGG and GO annotation. In addition, almost all *TPS* genes were highly co-expressed with TFs, accounting for one-third of the top 30 co-expression pairs. For instance, *ANT*, *AP2*, *MYB3*, *TCP*2 and *SPL12* are highly co-expressed with *TPS1*, *TPS12*, *TPS11*, *TPS8* and *TPS6* respectively. Furthermore, *WRKY21* and *WRKY16* are highly co-expressed with *FPS1* and *HMGR2* respectively (Fig 4).

### Sequencing of the sRNAs from four different tissues

**Fig 5:**
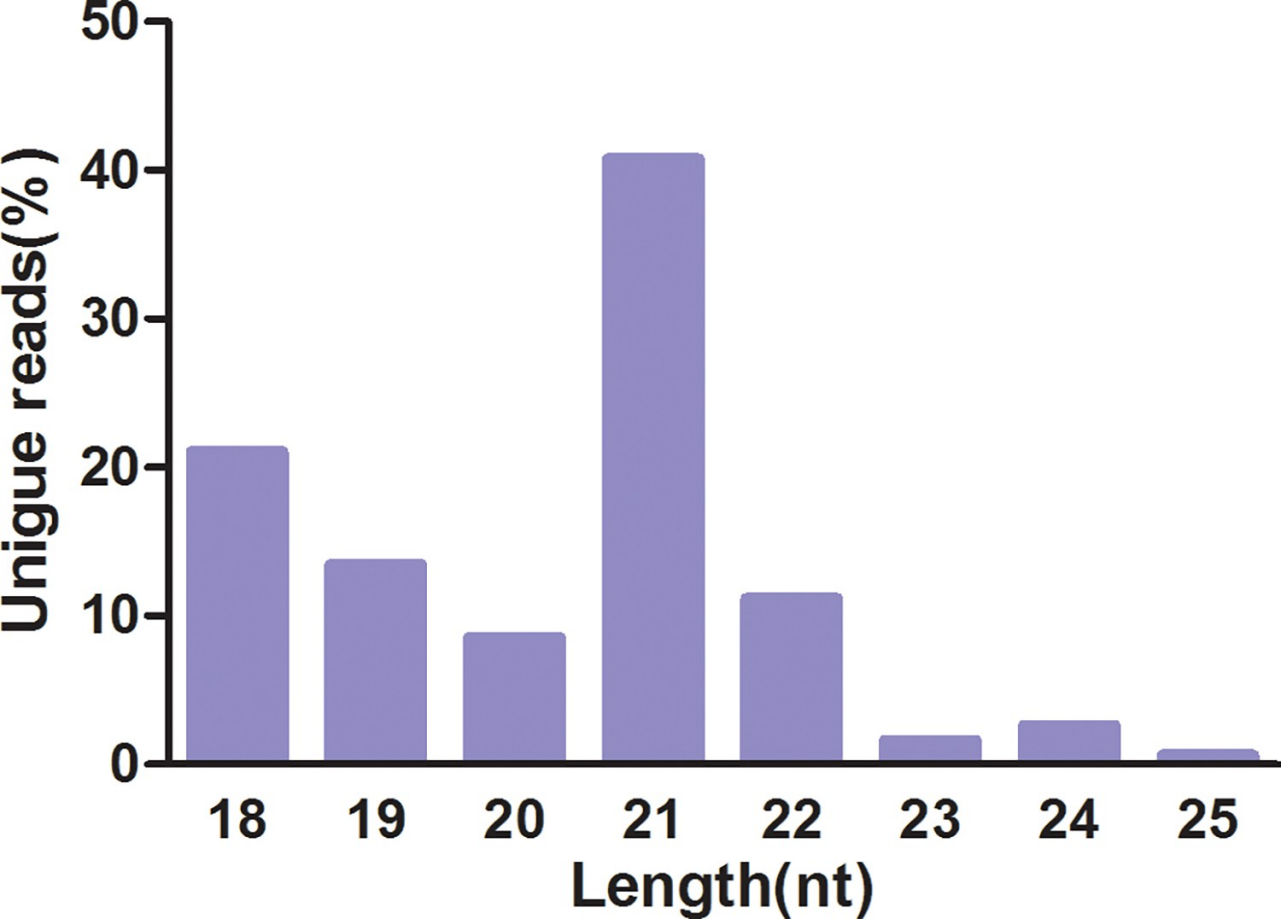
Length distributions of unique miRNAs.

To characterize the expression patterns of small RNA in *Paeonia*, small RNA libraries using the same samples from transcriptome analysis were constructed. After the removal of low-quality and small RNA reads derived from other small non-coding RNAs (including tRNA, snRNA, rRNA, snoRNA and Rfam RNA), 5207890, 7096767, 5736916, 5302298, 11097750, 11339971, 5770059, and 8525420 clean sRNA sequences were obtained, respectively (Table 1). Totally, we obtained 60077071 clean reads from all libraries. The size distribution analysis of unique sequences revealed that a large number of small RNAs ranged from 18nt to 24nt in size and the most abundant small RNAs was 21nt, which was in accordance with the length distribution of mature miRNA in other plants [52, 53] (Fig 5). The cloning frequencies of sRNAs with diverse sizes (15-32nt) were similar amongst the samples except for one root sample (S4 Fig). However, only a few sequences could be mapped to the transcriptome of *Paeonia* due to the absence of complete genome sequences.

### Identification of conserved and new miRNA

To identify the conserved miRNA in *Paeonia*, all the mappable sRNA sequences were first aligned with the already known plant precursor or the mature miRNA sequence in the miRNAs v21.0 database. Consequently, 237 pre-miRNAs corresponding to 257 candidate conserved miRNAs were identified in all, most of which were 18-22nt in length. The sequences of the miRNAs and pre-miRNAs were presented in S4 Table. Usually, miRNAs are significantly conserved among species. Thus, we also analyzed the miRNA conservation by calculating the frequency of *Paeonia* miRNAs found in other species. The identified conserved miRNAs were highly homogenous with the rest of other plants, like *Populus trichocarpa*, *Glycine max*, *Malus domestica*, *Zea mays*, *Oryza sativa*, *Manihot esculenta*, *Linum usitatissimum* and *Medicago truncatula*, where more than 100 miRNAs were homologous (S5 Fig, S5 Table). Among them, there are more than 100 homologous miRNAs, and the homology with *Glycine Max* was the highest with 221 homologous miRNAs.

**Fig 6:**
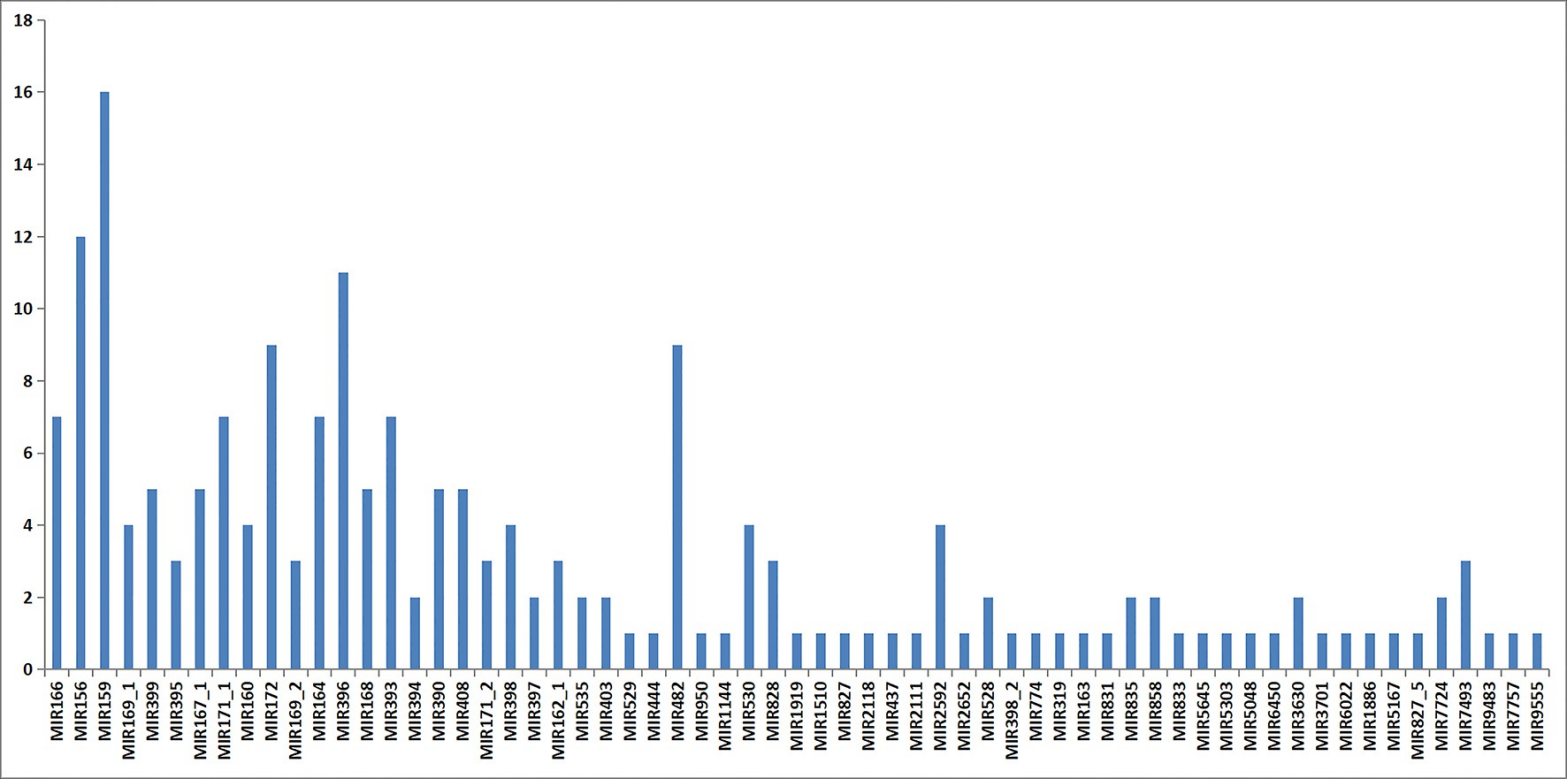
Quantity of distinct members presenting in conserved miRNA families in *Paeonia*.

Based on the sequence similarity, these candidates conserved miRNAs belonged to 63 miRNA families and the quantity of miRNA members in each miRNA family was significantly different (Fig 6). MiR159 served as the biggest miRNA family with 16 members, followed by miR156 with 12 members, miR396 with 11 members, miR172 with 9 members, and miR164/166/171/393 with 7 members each, whereas 29 miRNA families had only one member. Among them, most identified miRNA families such as miR156, miR160, miR166, miR171 and miR396 were highly conserved in various plant species, respectively. The most conserved miR156 was discovered in 49 species, followed by miR396 and miR166 which were identified in 45 and 42 plant species respectively. In addition, 29 known miRNAs such as miR437, miR444, miR744, miR845, miR894, miR1144, miR1886 and miR1919 were still categorized as non-conserved, as they were merely discovered in one or a few species.

Generally, the vital standard for the prediction of new miRNAs is whether the flanking sequence of miRNAs can fold back into a steady stem-loop structure. As per the current annotation standards of plant miRNAs, 13 pre-miRNAs corresponding to 22 new miRNAs were first identified in this study, of which most were 21nt in length (S4 Table). The minimum free energy (MFE) of those predicted pre-miRNAs miRNA precursors changed from -97.1kcal/mol to -25.4 kcal/mol with a mean of -60.34 kcal/mol, and the MFE index (MFEI) varied from 0.90 to 1.80 with an average of 1.12. When the MFEI is more than 0.85, the sequence is most likely to be miRNA [54]. The results aforementioned reach the steadiness demands of the secondary structure of miRNAs. The expression levels of those new miRNAs were different, and their normalized reads varied from 1 to 12229. PC-5p-97-25693, PC-3p-503-5584, PC-3p-122-21043 and PC-3p-96-26140 were the most abundant miRNAs.

### Differential expression of miRNAs from four tissues in *Paeonia*

**Fig 7:**
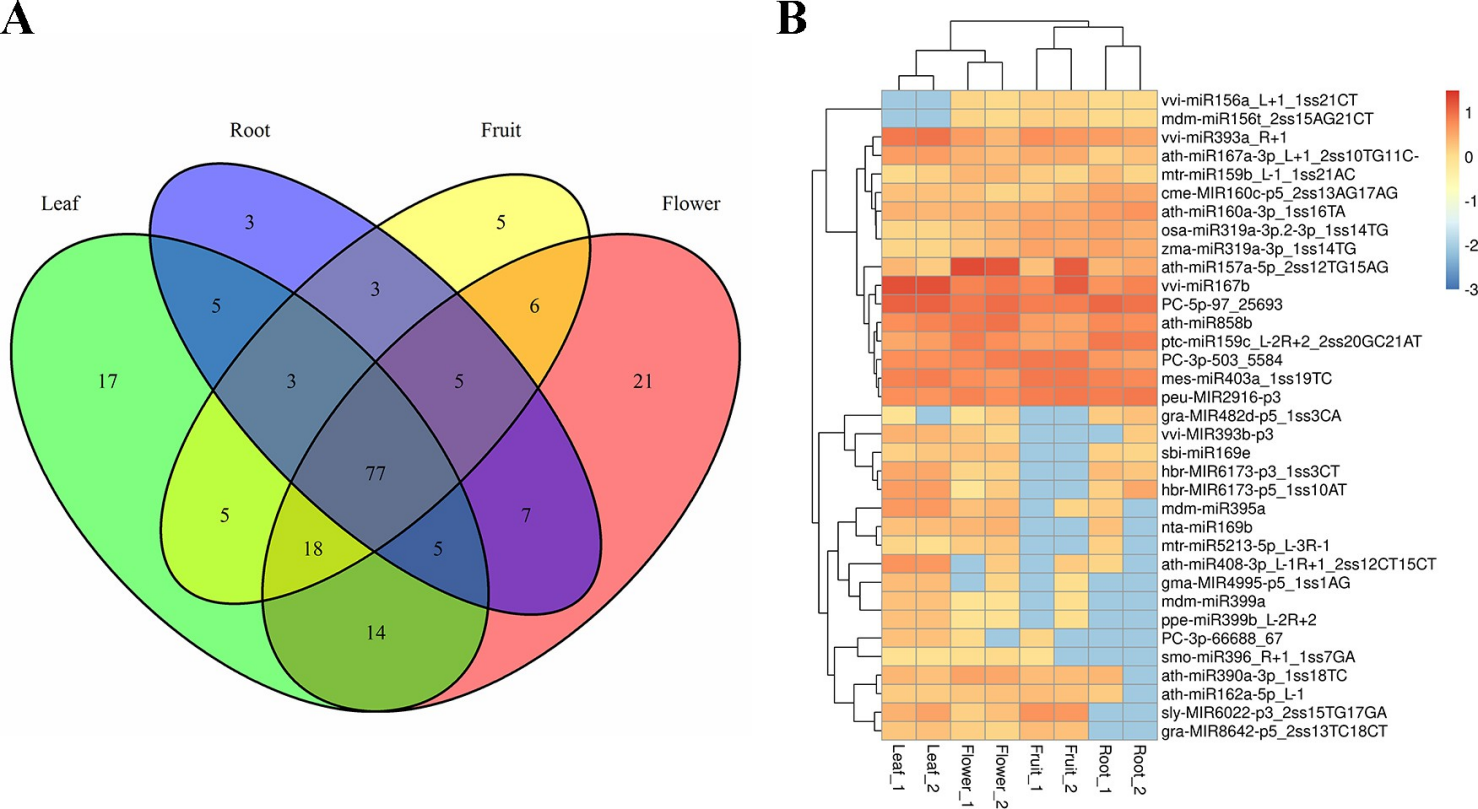
Tissue-specifically expressed miRNAs in *Paeonia*. (A The spatial expressing patterns of the miRNAs in four different tissues of *Paeonia*, B Differential expressing levels of miRNAs in four different tissues of *Paeonia*).

Among all the identified conserved and novel miRNAs, the numbers of the miRNAs at the intersections of two diverse tissues were calculated. As presented in Fig 7A, the largest number of miRNAs in common was identified between the leaves and flowers, revealing that the homogeneousness of the miRNAs between these two tissues is greater in contrast to the rest of tissue combinations. We also found that besides 77 miRNAs expressed in all tissues, 21 miRNAs were only expressed in flower, 17 miRNAs were only expressed in leaf, 5 miRNAs were only expressed in fruit, and 3 miRNAs were only expressed in root, and most of them exhibited a low expressing level. However, the majority of miRNA were expressed in two or three different tissues.

Based on the fold change (log2) among four tissue samples, a hierarchical cluster assay was performed on differentially expressed miRNAs (DEMs), and the miRNAs with identical or similar expressing features were clustered together. As shown in Fig 7B, 35 miRNAs in all, such as 32 known miRNAs and 3 new miRNAs displayed differential expression patterns. According to the heatmap of DEMs (Fig 7B), 8 out of 35 miRNAs exhibited highly expression in leaf, 5 out of 35 miRNAs exhibited highly expression in fruit, and 4 out of 35 miRNAs exhibited highly expression in root and flower, respectively. Moreover, 14 miRNAs showed comparatively low expression levels in four tissues. These miRNAs may cast a vital impact on the paeoniflorin and monoterpenoids biosynthesis of *Paeonia*. These outcomes offered a pivotal foundation for the exploration of miRNAs related to the paeoniflorin and monoterpenoids biosynthesis.

### Target identification by degradome analysis

To explore the underlying biological function of these identified miRNAs, a degradome sequencing method was adopted to identify the targets of *Paeonia* miRNAs. The sequence of miRNAs and their targets usually exhibit perfect complementarity in plants [55]. A total of 11773225 mappable reads were acquired from the degradome library of *Paeonia* (combination of root, stem, leaf and flower) (S6 Table). As per the relative abundance of target tags, the sliced-target transcripts identified by CleaveLand 3.0 were grouped into five categories (S7 Table) [56]. These different categories for targets were shown in the target plots (T-plots) (S6 Fig). A total of 65, 16, 396, 33 and 391 were identified and classified into categories 0, 1, 2, 3 and 4, respectively. More than half of the targets fall into categories 0–2, which indicates that most of these targets were efficiently cleaved by miRNA. Finally, 901 targets for 157 miRNAs (153 conserved and 4 new miRNAs) altogether were identified. The major group of targets was annotated as transcription factors, such as *MYB*, *bHLH*, *WRKY*, *NAC*, *SBP*, *TCP*, *SPL*, *AP2*, *ERF* and *bZIP*, etc. Most of them participate in the modulation of monoterpenoid biosynthesis in the plant according to previous studies [43, 57]. Furthermore, four new miRNAs target seven specific genes, including that PC-3p-122-21043 target three transcripts, and PC-3p-1283-2426 target two transcripts, while PC-3p-2538-1328 and PC-5p-18231-232 target one transcript.

### Annotation and enrichment analysis of targets for miRNAs

**Fig 8:**
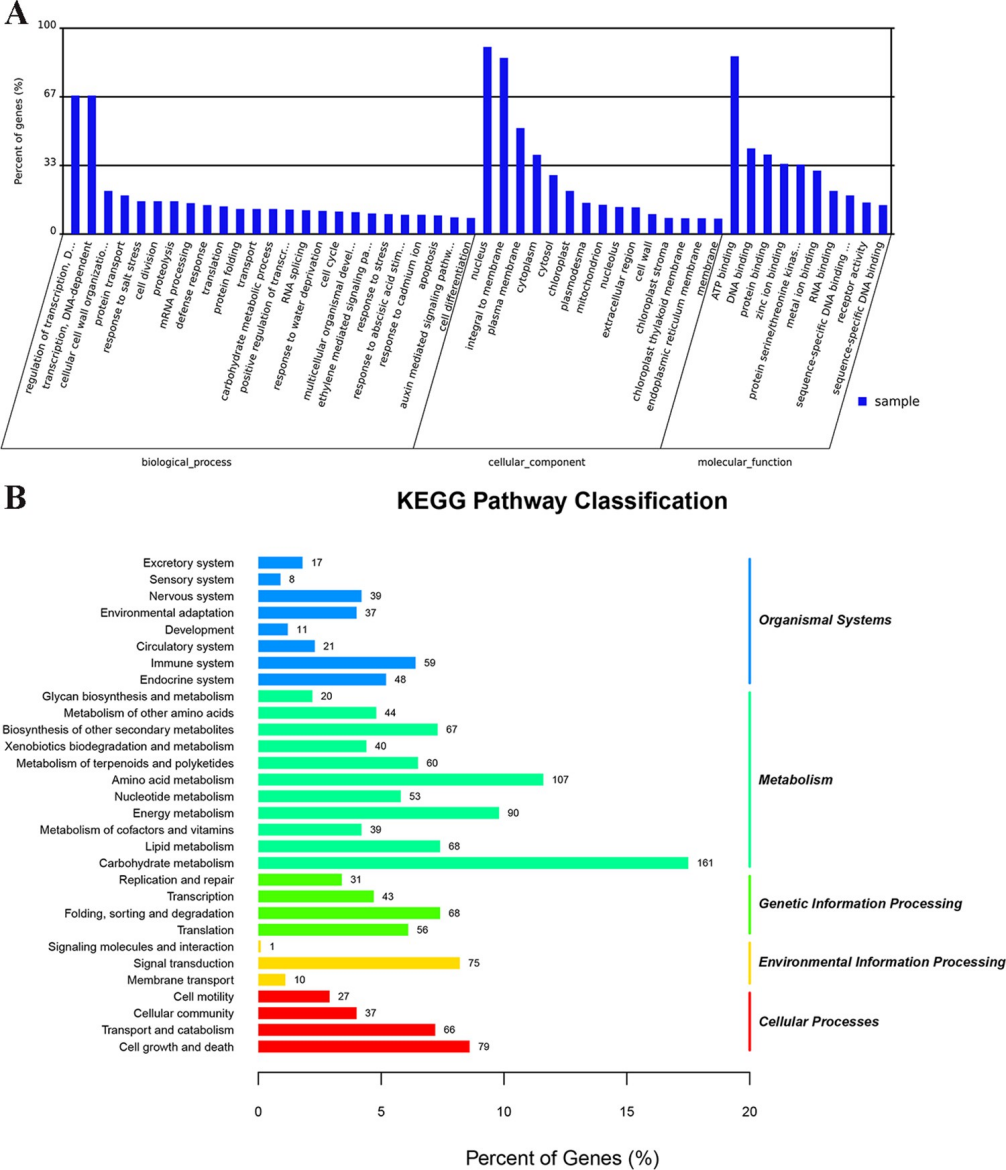
Annotation and enrichment analysis of targets for miRNAs. (A GO enrichment analysis of predicted targeted genes, B KEGG enrichment analysis of predicted targeted genes).

The GO enrichment analysis for all miRNAs targets was analyzed based on the GO database. In all, 34 GO terms were significantly enriched with a p-value less than 0.05, such as 20 biological processes and 14 molecular components terms (S8 Table). The GO function annotation indicated that the miRNAs target genes participated in a variety of molecular functions and biological processes (Fig 8A), such as the regulation of transcription, transcription, DNA-dependent, protein transport, transport, translation initiation factor activity, integral to membrance, ATP binding, nucleus, DNA binding, nucleus, protein binding, RNA metabolic process, and so on. These results showed that miRNAs played critical impacts on roles in the growth and development, metabolism, transport, response to biotic and abiotic stress, reproduction, and other processes of *Paeonia*.

The annotated pathways were achieved on the targets of miRNAs on the foundation of the KEGG database. There were 871 miRNA targets in total fell into 230 pathways (S9 Table). As shown in Fig 8B, the six pathways with the most abundant miRNA targets in metabolism were the metabolism of Carbohydrate, Energy, Amino acid, Lipid, and Terpenoids and Polyketides, as well as Biosynthesis of other secondary metabolites. There are also some miRNA targets abundant in the “Membrane transport”, “Transport and catabolism” and “Transcription” pathways. As shown in the S10 Table, nine miRNA targets were annotated in the main pathways for the paeoniflorin and monoterpenoids biosynthesis, including the terpenoid backbone biosynthesis pathway, monoterpenoid biosynthesis pathway, and limonene and pinene degradation pathway, while two miRNA targets were annotated in transport pathways. The miRNA targeting TFs were much more than that in the paeoniflorin and monoterpenoids biosynthesis and transport, reaching 32 miRNAs. Moreover, 75 of 169 the differentially expressed TFs were related to the biosynthesis and transportation of paeoniflorin and monoterpenoids, which suggested that miRNAs play straight and non-straight impacts on the genes participating in the biosynthesis and transport of paeoniflorin.

### Correlation analysis of miRNAs and their target genes

In the identified miRNA-target pairs, most of miRNAs cleaved two or more different transcriptional targets, while 33 miRNAs merely cleaved a single target transcript. In addition, it was found that different members of the miRNA families cleaved the same target gene or different members of a gene family, e.g., *CYP86A2* gene was cleaved by five miRNAs and two of them were in the same miRNA families. On the other hand, two or more targeted transcripts were identified for the identical miRNAs. Taking aly-miR835-p3 as an example, we could see that it could cleave 30 different transcripts, which participated in the process of transcription, signal transduction mechanisms, secretion and vesicular transport, RNA processing and modification, intracellular trafficking, energy production and conversion, secondary metabolites biosynthesis, transport and catabolism, etc. The result suggested that the modulation regulation activity of miRNAs in the life process was quite sophisticated.

**Fig 9:**
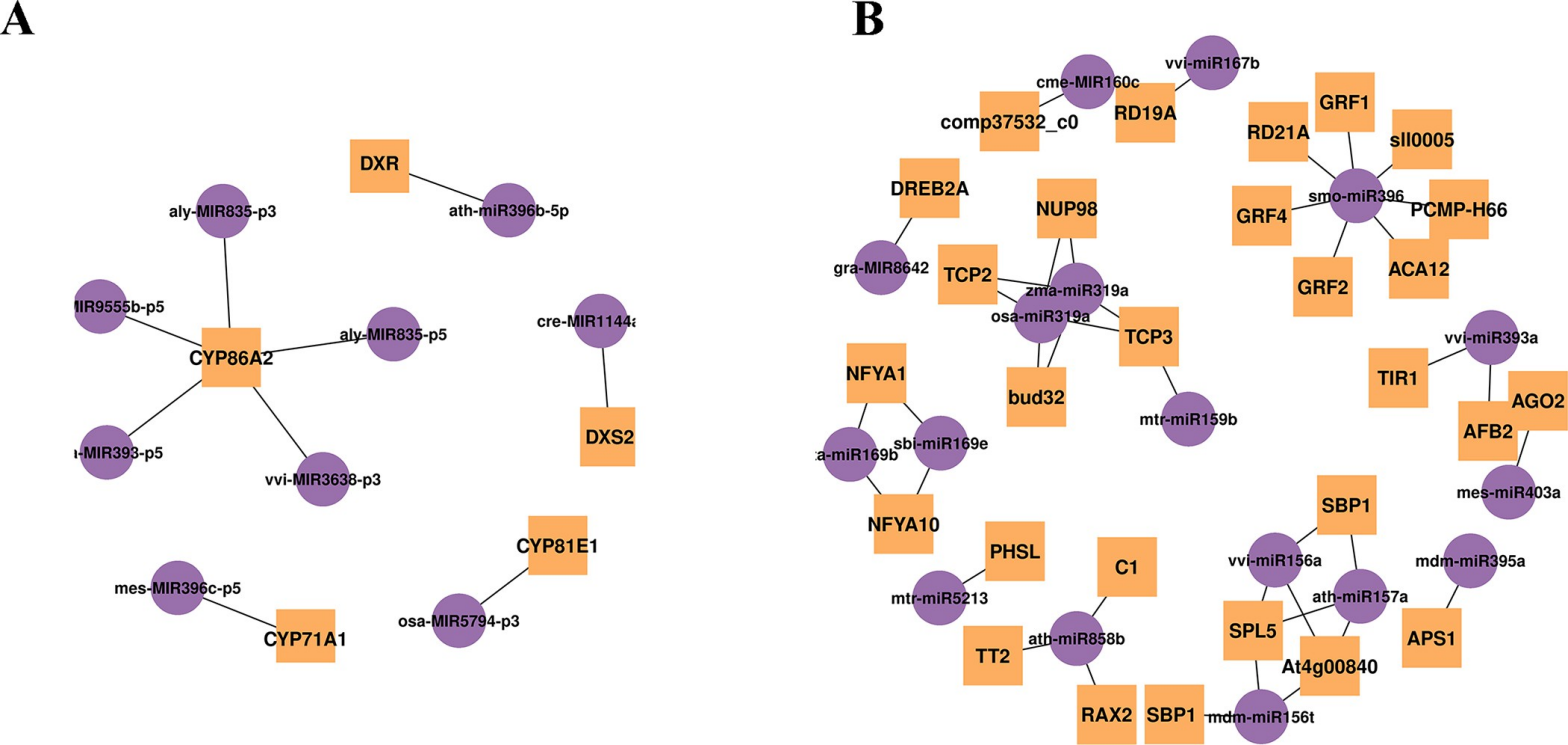
MiRNAs regulatory network. (A Network of relationship between miRNAs and targeted genes related to monoterpenoids biosynthesis, B Network of relationship between DEMs and targeted genes, Yellow squares represent target genes and purple circles represent miRNAs).

There are two strategies to screen for interested miRNA-mRNA interaction pairs in the study. On one hand, the DEGs in the monoterpenoids biosynthesis pathway were used as criteria to screen the corresponding DEMs (S11 Table). According to the degradome sequencing, only few monoterpenoids biosynthesis related DEGs could be targeted by miRNAs, and nine miRNA-mRNA pairs have been ultimately observed. Most of the corresponding miRNAs exhibited slightly differential expressing. The regulation network of miRNAs and the target genes in the monoterpenoids biosynthesis was constructed and listed in Fig 9A.

On the other hand, DEMs were used as screening criteria to obtain target genes (DEGs). The result showed that 17 out of 35 DEMs could match target genes according to the degradome sequencing and 41 miRNAs-mRNA pairs were observed eventually (S10 Table). The functional annotation of these screened DEGs showed that most of the DEGs regulated by DEMs were transcriptome factors and catalysis enzymes encoding genes, rather than the key enzyme genes in the paeoniflorin and monoterpenoids biosynthesis pathway. Among them, miR319a regulates transcription factors *TCP2* and *TCP3*, and miR159b regulates transcription factors *TCP3*, while miR858b regulates transcription factors *TT2*, *RAX2* and *MYBC1*, and miR157a regulates transcription factors *SPL5* and *SBP1*. The co-expression network analysis in the previous part shows that *TCP2*, *SPL12*, *MYBC1* and *AP2* are highly co-expressed with the key enzyme genes *TPS8*, *TPS6*, *ALDH2B7* and *CYP86A2* related to the terpene biosynthesis, suggesting that miRNAs indirectly regulate the paeoniflorin and monoterpenoids biosynthesis or participate in other physiological processes by regulating transcription factors. The regulatory network of DEMs and the targeted genes were presented in Fig 9B.

**Fig 10:**
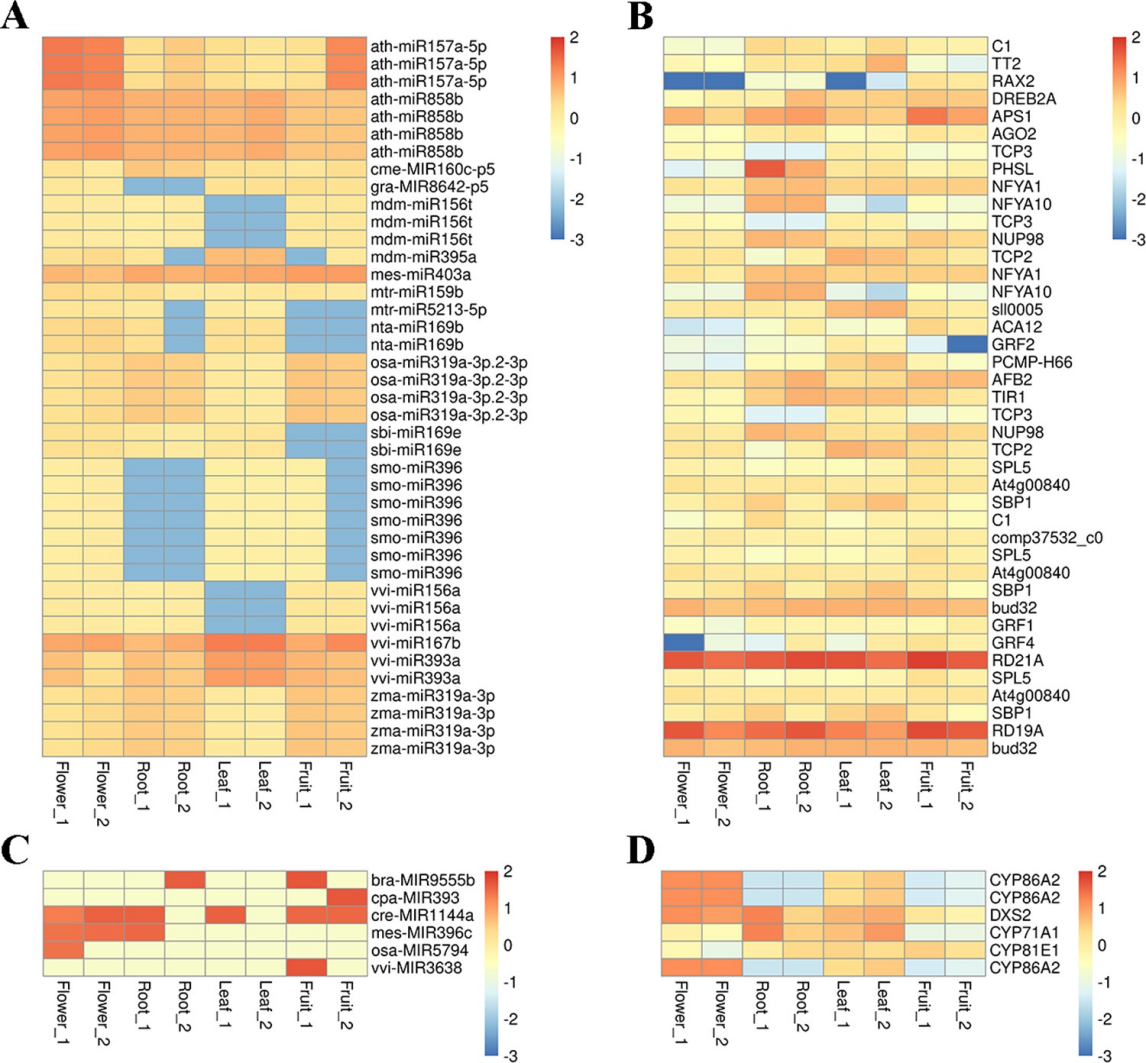
A combined views of the expressing level between miRNAs and their targets with differential expression in *Paeonia*. (A and B showed the one-to-one mapping correlation by DEMs in *Paeonia*, C and D showed the one-to-one mapping correlation by DEGs in monoterpenoids biosynthesis pathway of *Paeonia*).

All expression patterns of miRNA and corresponding targets aforementioned were showed in Fig 10A–10D by one-to-one mapping. It was easy to find that the expressing patterns of miRNA and mRNA in some miRNA-mRNA interactions were reversed in the identical tissues, which meant some miRNA exhibited a negative regulatory pattern. For instance, gra-miR8642-p5 was highly expressed when its target gene *NAFA1* was lowly expressed in the root, and bra-miR9555b-p5 and cpa-miR393-p5 were lowly expressed when their target gene *CYP86A2* were highly expressed in root and fruit. Conversely, certain miRNAs exhibited a positive regulation pattern. For instance, osa-miR319a-3p was highly expressed in the root and fruit, and its target genes *AFB2* and *TIR1* were also highly expressed in the same tissues. Here, we focus on the previously mentioned miRNA-mRNA interaction pairs associated with the monoterpenoid biosynthesis and transport, some of which directly regulate the related monoterpenoid biosynthesis genes, and some of which regulate the monoterpenoid biosynthesis genes via transcription factors. The analysis of DEGs results revealed that the expressing levels of those miRNAs were negatively associated with the expressing levels of the two TFs and their co-expression terpene biosynthesis genes. These outcomes enhance the effect of identified miRNAs on the monoterpenoid biosynthesis pathway.

### RT-qPCR validation of miRNAs and target genes

**Fig 11:**
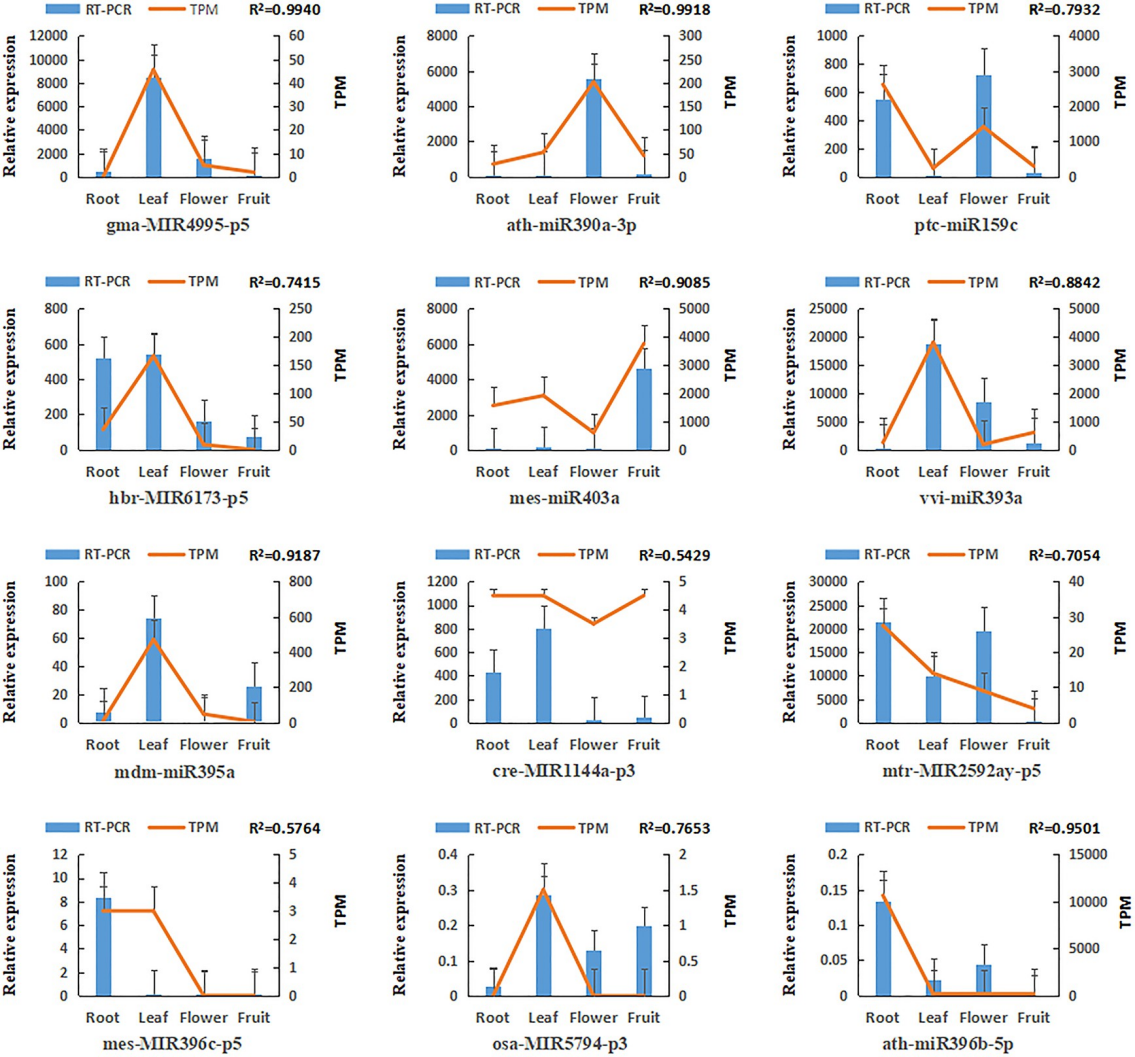
Expression levels of 12 miRNAs. (The bar charts and linear diagrams indicate the RT-qPCR and TPM data of the miRNAs, separately. The R^2^ value denotes the correlation between the RT-qPCR and TPM data).

**Fig 12:**
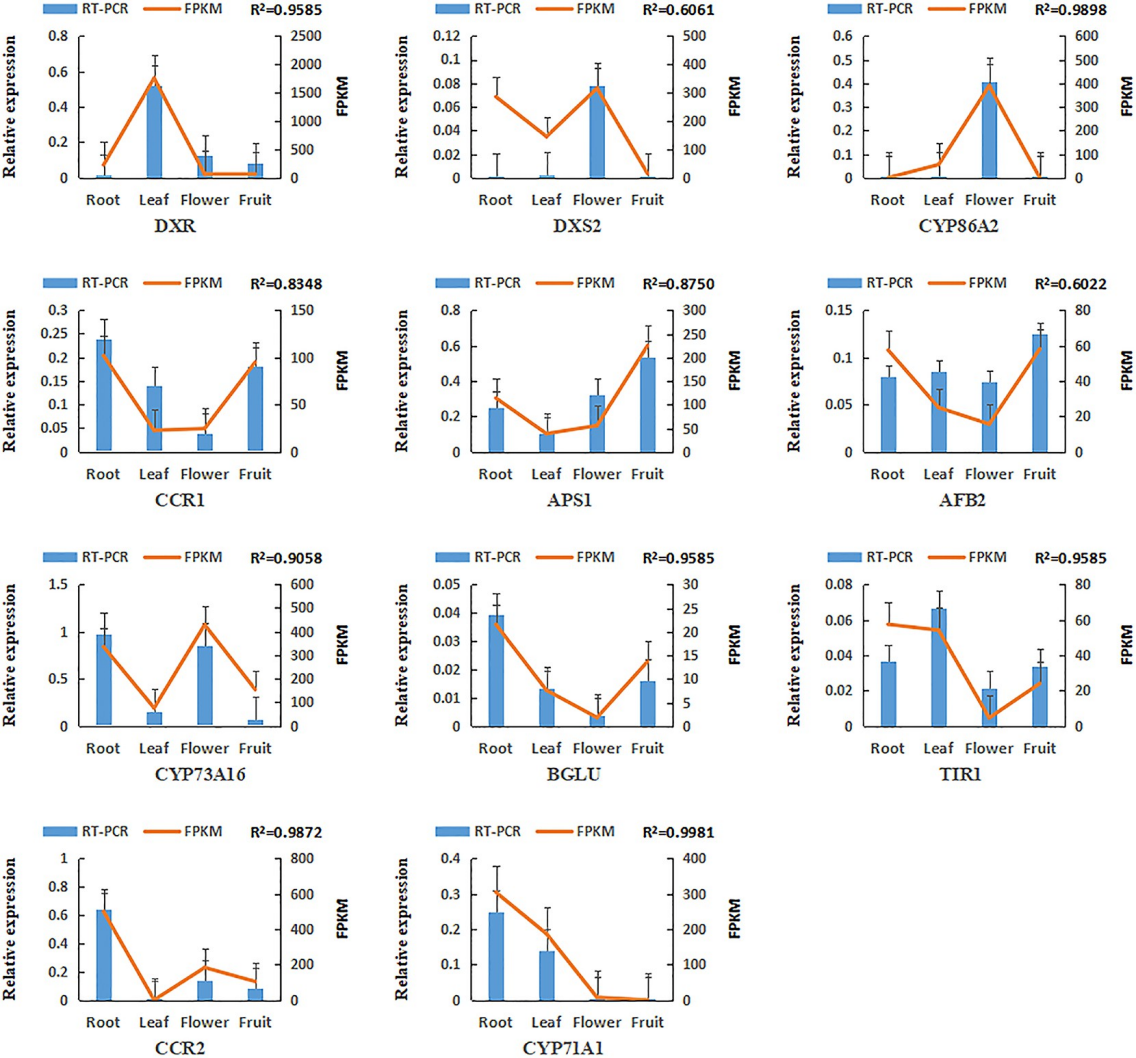
Expressing levels of 11 targeted genes. (The bar charts and linear diagrams indicate the RT-qPCR and FPKM data of the genes, separately. The R^2^ value denotes the correlation between the RT-qPCR and FPKM data).

To assess the variations in gene expression patterns on a functional level, 12 miRNAs and 11 target genes were screened for real-time quantitative PCR assay. The expressing data of the four tissues was acquired by RNA-sequence and RT-qPCR (Figs 11 and 12). The correlation between RNA sequence (FPKM/TPM) and qPCR (2^*-Δct*^) results for the miRNAs and genes was computed. As a result, the expressed pattern of most genes and miRNAs detected from RT-qPCR resembled that of RNA-seq. The correlative value (R^2^) was between 0.5429 and 0.9940 among the 12 miRNAs (Fig 11). For target genes, R^2^ of 11 genes was in the range of 0.5342–0.9585 (Fig 12). In Summary, the results revealed that the RNA-seq and RT-qPCR were in good consistency, indicating that small RNA and mRNA transcriptome sequencing had good reliability and accuracy.

## Discussion

### The tissue-specific accumulation pattern of monoterpene glycosides facilitates the identification of genes involved in biosynthesis and transport

The synthesis and accumulation of terpenoids were spatially and temporally specific in plants, varying in different tissues and at different development stages. This property was demonstrated in past researches [58, 59]. The high accumulation of paeoniflorin in the root implies that certain terpenoid biosynthesis-related genes might exhibit remarkable expression in the root, or may be synthesized in other tissues and transported to the root for accumulation. The tissue-specific accumulation pattern of paeoniflorin facilitated us to investigate the molecule mechanism of its biosynthesis and transport. At present, as an important complement to secondary metabolites biosynthesis, numerous studies have shown that a transporter-based transport mechanism is involved in the process of plant accumulation [60, 61]. ABC transporter, the most important transporter discovered so far, plays a vital regulatory effect on the accumulation of secondary metabolites such as alkaloids, terpenes and phenols in plants [62–64]. Here, we found that 69 transporter-related genes were differentially expressed in four tissues, most of which were ABC transporters, although the specific biological function of these transporter genes is unknown. In this study, only two miRNAs, gma-miR4388-p3 and cme-miR172d-p3, were predicted to target transporter genes, *ABCC2* and *TGD1*respectively. As one of the earliest cloned and characterized ABC transporters, *ABCC2* transporter transports glutathione conjugates, glucuronide conjugates and chlorophyll catabolites [65–67]. Further researches revealed that *ABCC2* transportseven more substrates, and is vital for polar auxin and secondary metabolite transport, lipid degradation, detoxification of exogenous toxins, plant disease resistance and stomatal function regulation [68, 69]. *TGD1*, together with *TGD2* and *TGD3*, forms a putative ABC transport complex for lipid transportation, in which *TGD1* acts as an ABC transporter permease [70]. Currently, relatively little is known regarding the transporters and mechanisms underlying the transport activity of paeoniflorin and other monoterpene glycosides. Thus, we focus on the biosynthesis in this paper, and the identification of specific transporters of monoterpene constituents and the transport mechanism will be further study in the future.

### Transcriptome analysis as a powerful tool for the discovery of target genes

Research of terpenoids biosynthetic pathway and the related genes has progressed with the development of molecular biology and genomics. Transcriptome analysis as a powerful tool for the discovery of target genes. Yuan et al. [6] has identified 19 EST sequences related to the paeoniaflorin biosynthesis in terpenoid backbone pathway by using sequence homology. They also focused on function diversity of these genes. Lu et al. [49] found 32 genes related to terpenoid and monoterpenoid glycoside biosynthesis and one gene encoding (-)-α-terpineol synthase through comparative transcriptome analysis of shoots of different strains (with different paeoniflorin content) in *Paeonia*. They uncovered that were the differences in terpenoid biosynthesis ability among different materials may be largely caused by *ACAT1*, *HMGS*, *MK*, *PMK*, *GGR*, *FPS1 and MVD* genes. However, a series of subsequent processes after the synthesis of terpenoid backbone were not fully understood. In our study, through the comparison transcriptome analysis of four different tissues, mining the terpenoid backbone biosynthesis related genes, we also screened the monoterpenoids biosynthesis pathway, and the limonene and pinene degradation pathway in order to obtain more comprehensive paeoniflorin biosynthesis related genes. Ultimately, a total of 115 related DEGs were screened out, including 30 terpenoid backbone biosynthesis related genes, 13 *TPS* genes and 72 *CYP450* genes, which were all key enzyme genes in the paeoniflorin and monoterpenoids biosynthesis process. After the synthesis of monoterpenoids by *TPS*, the enzymes encoded by these genes aforementioned played important post-modification role through a series of activities of hydroxylation, glycosylation, methylation, isomerization and epoxidation [71]. Although the specific functions and subcellular localization of most genes in the paeoniflorin and terpenoid biosynthesis pathways remain to be further identified and verified, there is still a certain expansion compared with previous studies by Yuan et al. [6] and Lu et al. [49]. In addition to mining genes related to monoterpene glycoside biosynthesis by comparing transcriptome sequencing results of different tissues, we also profiled the miRNA expression among different tissues and revealed the regulation interaction of miRNA on monoterpene glycoside biosynthesis genes by small RNA and degradome sequencing.

### MiRNA regulates genes involved in monoterpene glycoside biosynthesis and transport

Several miRNAs from *Paeonia* have been screened and characterized by high throughput sequencing, which participated in the regulation of the yellow formation in flowers [34] and response to *Botrytis cinerea* [33]. Nevertheless, up to now, the research on miRNAs involved in terpenoid biosynthesis is scare. Recently, researchers [6, 49] performed comparative transcriptomics analysis to identify candidate genes associated with the terpenoid and paeoniflorin biosynthesis in *Paeonia*. However, transcriptome sequence alone couldn`t thoroughly elucidate the genetic regulation network involved in the terpenoid and paeoniflorin biosynthesis. The combination of multi-sequencing is a universal method to analyze the various mechanisms of plant life-cycle, such as the anthocyanin biosynthesis in sweet potato [32] and the salt tolerance in Sesame [36]. In the present study, we employed transcriptome, small RNA and degradome sequence for the first time to elucidate the paeoniaflorin and monoterpenoids biosynthesis processes in *Paeonia*.

**Fig 13:**
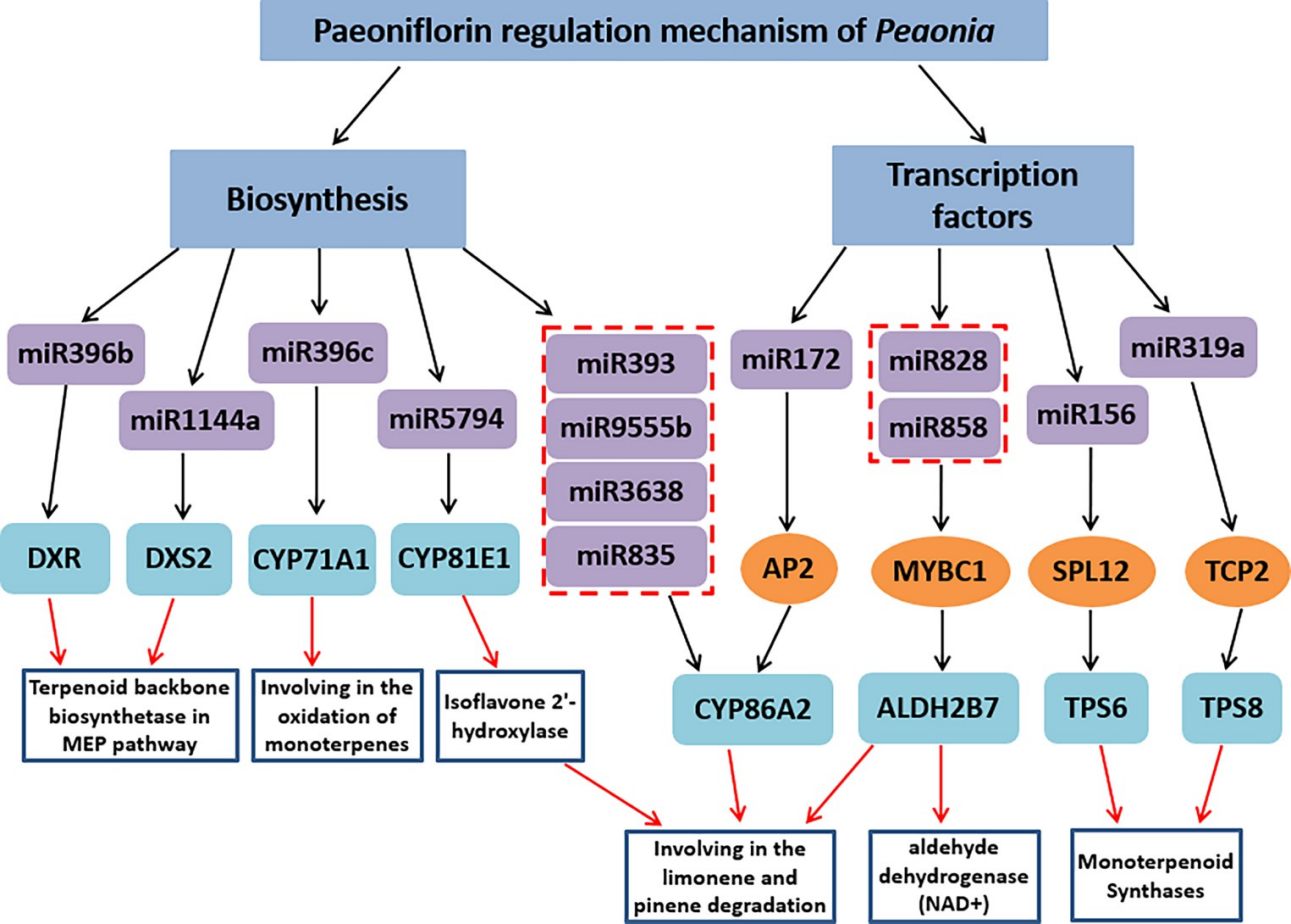
Paeoniflorin regulation mechanism of *Paeonia*.

Herein, miRNAs associated with the regulation of the genes involved in terpenoid backbone biosynthesis, monoterpenoid biosynthesis, limonene and pinene degradation were explored. The whole regulation network was shown in Fig 13. MiR1144a and miR396b putatively regulated *DXS* and *DXR* from MEP pathway, respectively. *DXS* is the rate-limiting enzyme of MEP pathway. In *Arabidopsis thaliana*, up-regulating or down-regulating the expression of *DXS* gene could affect the contents of chlorophyll, tocopherol, carotenoid, gibberellin and abscisic acid [72]. In addition, miR835-p5, miR835-p3, miR9555b, miR393 and miR3638 putatively regulated *CYP86A2* enzyme gene, while miR396c and miR5794 regulated *CYP71A1* and *CYP81E1* enzyme gene, respectively. Genes from the *CYP71A* subfamilies have been associated with the oxidation of monoterpenoids in different plant species of various families [73–75]. *CYP71A1* demonstrated its hydroxylation ability of the monoterpenoids (nerol and geraniol) [76, 77]. *CYP86A2* was a cytochrome P450 monooxygenase that catalyzed fatty acid oxidation, exerting a primary effect on the biosynthesis of extracellular lipids in *Arabidopsis* [78]. Several members of the *CYP81E* subfamily catalyzed the hydroxylation of isoflavones, daidzein and formononetin. *CYP81E1* was an isoflavone 2`-hydroxylase, which was identified in *Callerya speciose* and *Glycyrrhiza echinata* [79, 80]. Although the functions of *CYP86A2* and *CYP81E1* were not necessarily associated with monoterpene biosynthesis in other plants according to the reports aforementioned, we still regard these two enzymes as part of the paeoniflorin biosynthesis pathway, since *CYP450* enzymes had heterogenous substrates and their functions varied between plants.

### MiRNAs indirectly regulate the monoterpenoid biosynthesis genes via TFs

In addition to the above miRNAs that directly regulate the related monoterpene biosynthesis genes, some miRNAs that indirectly regulate the monoterpenoid biosynthesis genes via TFs were also explored through the co-expression network analysis (Fig 13). Five miRNAs including miR172, miR828, miR858, miR319 and miR156 putatively regulated four TFs namely *AP2*, *MYBC1*, *TCP2* and *SPL12* respectively. Previous studies have reported that TCP genes such as *TCP2*, *TCP3*, *TCP4*, *TCP10* and *TCP24* in Arabidopsis have miR319a binding site [81]. The results of our study were also consistent with previous results, where TCP2 could also combine with miR319a. MiR319a has been proven to have cleavage activity on *AtTCP* transcripts. Class II members of TCP transcription factors (CIN) played an inhibitory effect in terms of cell proliferation in leaves [82]. TCP proteins with high or low levels of miR319a binding sites may trigger excessive cell proliferation, resulting in leaf enlargement in the *Arabidopsis* and tomatoes [83]. The co-expression network results showed that *TCP3* was highly co-expressed with the monoterpene synthase gene *TPS2*, suggesting that miR319a regulated the expression of *TPS2* via transcription factor *TCP3*.

### TFs regulating the monoterpene biosynthesis

In recent years, some transcription factors regulating the monoterpene biosynthesis have also been identified successively. For example, *AaNAC1*, *AaNAC2*, *AaNAC3*, *AaNAC4* and *AcEIL1*, *AcEIL2*, *AcEIL3* and *AcEIL4a* in *Actinidia* can positively regulate *AaTPS1* and promote the synthesis of monoterpenes (laurene, limonene and terpene) [84]; In sweet orange, the AP2 / ERF family transcription factor *CitAP2*.*10* can facilitate the synthesis of juluan sesquiterpene by binding to the promoter of the terpene synthase gene *CsTPS1* [85]. *MsMYB* reduces the content of sesquiterpenes (citrus oleene, gemasene and yilanolene) and monoterpenes (terpene) by inhibiting the expression of transcription factor *MsGPPS*.*LSU* (geranyl diphosphate synthase) in *Mentha spicata* [86]. It is worth noting that in the whole regulatory network we construct, the interaction between TFs and TPs query to the corresponding interaction in *Arabidopsis thaliana*. Some of the gene functions in biological processes, especially those involved in specialized of paeoniflorin and monoterpenoids, may be different in *Paeonia*. *Paeonia* is phylogenetically distant from *Arabidopsis thaliana*, despite being the closest species among the nine species in GeneMANIA database, which affects the accuracy and reliability of gene function predictions.

### Terpenoid-related miRNA-mRNA regulation modules

To date, a variety of terpenoid-related miRNA-mRNA regulation modules have been detected in other plant species. They regulate the biosynthesis of terpenoids via targeting genes that encode enzymes and TFs participating in the biosynthetic pathways. For instance, miR838, miR2919, miR5021, miR5251 and miR5658 in *Ferula gummosa* were predicted to regulate terpenoid biosynthesis-related genes encoding *MECT*, *ACAT*, *HMGR*, *FPS*, *DXS*, and *SQS*. MiR1533, miR5021 and miR5658 in *F*. *gummosa* were predicted to regulate terpene associated TFs genes, i.e. *SPL7*, *SPL11* and *ATHB13*, separately [43]. Nine *Xanthium strumarium* miRNAs, such as miR1134, miR5021, miR5183, miR5255, miR5491, miR6435, miR6449, miR7539 and miR7540, were forecasted to modulate terpene biosynthesis-related genes encoding *HMGR*, *IDS*, *IDI*, *GA3OX*, *squalene epoxidase*, *βAS*, *GAO*, *entkaurene synthase*, *DXS*, *and R-linalool synthase* [87]. In *Mentha spp*., miR156, miR414 and miR5021 were forecasted to realize the cleavage of the transcripts of genes that encode terpenoid biosynthesis-associated *DXS*, *TPS21*, *GGPPS*, and *IDI* [88]. In *Panax notoginseng*, miR5021, miR5293, miR5163 and new-miR-27 were forecasted to target *HMGS*, *geranyldiphosphate synthase* and *DXS* [89]. In *Eucommia ulmoides*, *DXS* and *GPS* were targeted by Eu-miR91 and Eu-miR15, respectively [56]. Even though the miRNAs aforementioned have been predict to target transcripts related to the terpenoids production, more experiment verification is demanded for confirmation of their regulatory functions. The confirmation could be completed via production and assay of transgenic plants with upregulation and downregulation miRNAs. This is one of the research works we will carry out in the future.

However, the study still had some limits. All tissues were only collected once, and the temporal component and plant developmental stage have not been considered in the study. The biosynthesis of paeoniaflorin and monoterpenoids may be triggered before our sampling, thus affecting the reliability of the results. To draw reliable conclusions, more than two replicates are needed. Another limitation of this study is that the functions and annotations of genes and miRNAs are based on other species. The role of specific genes and miRNAs in the biosynthesis of paeoniflorin and related monoterpenoids needs to be further investigated in Paeonia through gene silencing and knockout experiments.

## Conclusion

In conclusion, 289 miRNAs and 30177 unigenes were identified, of which nine miRNAs from seven miRNA families including miR396, miR393, miR835, miR1144, miR3638, miR5794 and miR9555 were verified to regulate the paeoniaflorin biosynthesis genes directly by degradome sequencing. The co-expression network analysis between the identified TFs and TPs showed that a total of 75 TFs and 32 TPs were involved in the co-expression network. Moreover, four monoterpenoid-regulating TFs namely *AP2*, *MYBC1*, *SPL12* and *TCP2* were putatively regulated by five miRNAs including miR172, miR828, miR858, miR156 and miR319 respectively. These miRNA-mediated TFs were exhibited remarkable co-expression with four genes including *CYP86A2*, *ALDH2B7*, *TPS6* and *TPS8*, which were involved in the monoterpenoid biosynthesis pathway. The present research is the first try in combining mRNA and miRNA expressing data with degradome analysis to reveal the tissue-specific regulation network of miRNAs and their targets in *Paeonia*, and it thoroughly analyzed the complex network of the paeoniaflorin and monoterpenoids biosynthesis. Those outcomes will improve the knowledge of the molecular mechanisms of paeoniaflorin biosynthesis mediated by miRNA to a new level, and provide a valuable resource for further study on *Paeonia*.

## Supporting information

S1 Table Primers for miRNA determination by RT- qPCR. (DOCX)

S2 Table Primers for targeted genes determination by RT-qPCR. (DOCX)

S3 Table The top 30 co-expression relationship according to the normalized weight. (DOCX)

S4 Table The entire expressed miRNA. (XLSX)

S5 Table Conservation profile of the identified miRNAs. (XLSX)

S6 Table Overview of degradome-seq results from raw data to the sequences of mapping. (XLSX)

S7 Table Target identification and expression analysis by degradome-seq. (XLSX)

S8 Table GO enrichment analysis of predicted targeted genes in *Paeonia*. (XLSX)

S9 Table KEGG enrichment analysis of predicted targeted genes in *Paeonia*. (XLSX)

S10 Table One-to-one mapping correlation by DEGs in paeoniaflorin biosynthesis pathway of *Peaonia*. (XLSX)

S11 Table One-to-one mapping correlation by DEMs in *Peaonia*. (XLSX)

S1 Fig BUSCO analysis for assessing transcriptome assembly and annotation completeness. (PDF)

S2 Fig GO enrichment analysis of DEGs between different tissues. (DOCX)

S3 Fig KEGG enrichment analysis of DEGs between different tissues. (DOCX)

S4 Fig Length distribution of counts of overall and unique sRNAs in this study. (PDF)

S5 Fig Conservation Profile of the identified miRNAs. (TIF)

S6 Fig T-plots for miRNA targets in the 5 diverse catagories verified by degradome sequencing. (DOCX)

S1 File Trinity transcript. (FASTA)

S2 File Differential gene expression analyses. (ZIP)

S3 File GO term enrichment analyses. (ZIP)

## Abbreviation

GPP: isoprene pyrophosphate
MVA: mevalonate
DXP: 1-deoxyd-dxylose-5-phosphate
MEP: methylerythritol-4-phosphate
HMGS: hydroxymethylglutaryl-CoA synthase
HMGR: 3-hydroxy-3-methylglutaryl-CoA reductase
MVK: mevalonate kinase
PMK: phosphomevalonate kinase
DXS: 1-deoxy-D-xylulose-5-phosphate synthase
DXR: 1-deoxy-D-xylulose 5-phosphate reductoisomerase
IspD: 2-C-mehyl-D-erythritol 4-phosphate cytidyltransferase
IspE: 4-diphosphocytidyl-2-C-methyl-D-erythritol kinase
IspF: 2-C-mehyl-D-erythritol 2,4-cyclodiphoaphate synthase
HDS: 1-hydroxy-2-methyl-2-(*E*)-butenyl 4-diphosphate synthase
HDR: 1-hydroxy-2-methyl-2-(*E*)-butenyl-4-diphosphatereductase
IDI: isopentenyl diphosphate isomerase
bZIP: basic leucine zipper
bHLH: basic helix-Loop-helix
SPL1: squamosa promoter binding protein-like gene
BUSCO: Benchmarking Universal Single-Copy Orthologs
Nr: Non-redundant
Pfam: Protein family
KEGG: Kyoto Encyclopedia of Genes and Genomes
KOG: eukaryotic Orthologous Groups
GO: Gene Ontology
RPKM: Reads Per Kilobase per Million mapped reads
BH: Benjamini-Hochberg
FDR: false discovery rate
FC: fold change
DEGs: differentially expressed genes
TFs: transcription factors
TPs: monoterpenoid biosynthesis related genes
MFE: minimum free energy
DEMs: differentially expressed miRNAs
SQS: squalene synthase
IDS: isopentenyl diphosphate/dimethylallyl diphosphate synthase
IDI: isopenteyl diphosphate isomerase
ACAT: acetyl CoA-acetyltransferase
FPS: farnesyl diphosphate synthase
GA3OX: gibberellin 3-oxidase
βAS: beta-amyrin synthase
GAO: germacrene A oxidase
MECT: 2-C-methyl-D-erythritol 4-phosphate cytidylyltransferase
