# Supplementary material for: A tissue-specific profile of miRNAs and their targets related to paeoniaflorin and monoterpenoids biosynthesis in Paeonia lactiflora Pall. by transcriptome, small RNAs and degradome sequencing
Source: PLoS One. 2023 Jan 26;18(1):e0279992. doi: 10.1371/journal.pone.0279992 (PMC9879538; doi:10.1371/journal.pone.0279992)
Supplement: S1 Table — (DOCX) [file pone.0279992.s001.docx]

S1 Table Primers for miRNA detection by RT-qPCR

| miRNAname | Prime Sequence (5`→3`) |
| --- | --- |
| mdm-miR395a | CTGAAGTGTTTGGGGGAACTC |
| vvi-miR393a | TCCAAAGGGATCGCATTGATCC |
| gma-MIR4995-p5 | GACTCATAGGCAGTGGCTTGGTT |
| mes-miR403a | TTAGATTCACGCACAAACCCG |
| hbr-MIR6173-p5 | GTAGTCCTATGCCGTAAACGA |
| ath-miR390a-3p | CGCTATCCATCCTGAGTCTCA |
| ptc-miR159c | TGGAGTGAAGGGAGCTCCTGA |
| mes-MIR396c-p5 | CGCGCGCTTCTTCTTCTTCATCTT |
| mtr-MIR2592ay-p5 | CGCCGAGTCGGGTTGTTTGGGA |
| cre-MIR1144a-p3 | CGTGCTGGTGGTGGTGGTGG |
| ath-miR396b-5p | CGCGCGCTTCCACAGCTTTCTTGATT |
| osa-MIR5794-p3 | GCCGCGCGTCTCCTTCAAGCTTATTC |
| U6-Forward | GGGGACATCCGATAAAATT |
| U6-Reverse | TGTGCGTGTCATCCTTGC |
