# Supplementary material for: A tissue-specific profile of miRNAs and their targets related to paeoniaflorin and monoterpenoids biosynthesis in Paeonia lactiflora Pall. by transcriptome, small RNAs and degradome sequencing
Source: PLoS One. 2023 Jan 26;18(1):e0279992. doi: 10.1371/journal.pone.0279992 (PMC9879538; doi:10.1371/journal.pone.0279992)
Supplement: S2 Table — (DOCX) [file pone.0279992.s002.docx]

S2 Table Primers for target genes detection by RT-qPCR

| Target genes name | Primer sequence (5`→3`) |
| --- | --- |
| DXR-F | AACTTTGCCTCGCCTAGACCTTTG |
| DXR-R | GAACCCCAGTCATAGTCCCTCCAG |
| DXS2-F | AGGAAGGGTGCTAAAGGAGGGAAG |
| DXS2-R | AGCCACGGTTATTGAGATGCCAAG |
| CYP86A2-F | TCGGTAGCTTGCCTGGTCTAGTC |
| CYP86A2-R | CTTGCGTGCCAAAAGCGGAATG |
| CCR1-F | GATGGAGCCAAGGAGCGACTTC |
| CCR1-R | GTTCTGCCTGTGGATCTGTGACTG |
| APS1-F | TGATACCCGTCGCCGCCTTC |
| APS1-R | AACGCCATCTTCGAGCACCTTC |
| AFB2-F | TCAAGGTGGAGCGATGGAGTAGG |
| AFB2-R | AAGGGCTTTAACCCGTGGAAACC |
| CYP73A16-F | CTTGAACCACCGCAACCTAGCC |
| CYP73A16-R | TGACGAAACCACCACGAGATTACG |
| BGLU-F | GTCAAGGCGTGGCGGATGTC |
| BGLU-R | TCCAAAACCGAAGGGGAAAAGAGG |
| TIR1-F | GCCGCATTTCGCTGACTTCAATC |
| TIR1-R | GGTACGCCTTCGCCATCTCAAC |
| CCR2-F | TGCGATGCCGAAACCGTGTG |
| CCR2-R | GACGACGTAGCCATGTTCCAGTAG |
| CYP71A1-F | TTGGCTCCTCGTGAATCAACTGC |
| CYP71A1-R | TCTCTCTGGGATGAACTCCAAGGG |
| Plactin-F | TTGTGCTGGATTCTGGTGATGGTG |
| Plactin-R | AGACGGAGGATAGCGTGAGGAAG |
