# Supplementary material for: A tissue-specific profile of miRNAs and their targets related to paeoniaflorin and monoterpenoids biosynthesis in Paeonia lactiflora Pall. by transcriptome, small RNAs and degradome sequencing
Source: PLoS One. 2023 Jan 26;18(1):e0279992. doi: 10.1371/journal.pone.0279992 (PMC9879538; doi:10.1371/journal.pone.0279992)
Supplement: S3 Table — (DOCX) [file pone.0279992.s003.docx]

S3 Table The top 30 co-expression relationship according to the normalized weight

| TFs | TPs | Normalized weight | TFs | TPs | Normalized weight |
| --- | --- | --- | --- | --- | --- |
| MYBC1 | ALDH2B7 | 0.002117 | MYB5 | CYP81F1 | 0.001335 |
| ANT | TPS1 | 0.002001 | AP2 | TPS12 | 0.001326 |
| TPS5 | TPS1 | 0.001943 | DXR | ALDH311 | 0.001323 |
| ISPD | ALDH3F1 | 0.001921 | MYB44 | TPS7 | 0.001311 |
| MYB3 | TPS11 | 0.001787 | ERF5 | CYP86A1 | 0.001302 |
| AP2 | CYP86A2 | 0.001728 | MYB305 | CYP82C4 | 0.001278 |
| ERF034 | CYP86B1 | 0.001679 | MYB48 | CYP76C4 | 0.001241 |
| MYB39 | CYP86B1 | 0.001664 | BHLH66 | ALDH3H1 | 0.001222 |
| TCP2 | TPS8 | 0.001583 | BHLH30 | TPS5 | 0.001212 |
| WRKY21 | FPS1 | 0.001581 | SPL12 | TPS6 | 0.001209 |
| ERF21 | CYP81F1 | 0.001440 | MYB44 | TPS8 | 0.001197 |
| TPS11 | TPS8 | 0.001438 | SPL9 | TPS1 | 0.001196 |
| RAP2-11 | CYP76C4 | 0.001403 | BHLH66 | CYP81D1 | 0.001164 |
| MYB21 | CYP86A1 | 0.001388 | ERF061 | TPS6 | 0.001149 |
| WRKY16 | HMGR2 | 0.001347 | TPS8 | TPS6 | 0.001143 |
