# Supplementary figures and images for: A tissue-specific profile of miRNAs and their targets related to paeoniaflorin and monoterpenoids biosynthesis in Paeonia lactiflora Pall. by transcriptome, small RNAs and degradome sequencing
Source: PLoS One. 2023 Jan 26;18(1):e0279992. doi: 10.1371/journal.pone.0279992 (PMC9879538; doi:10.1371/journal.pone.0279992)

# BUSCO Assessment Results

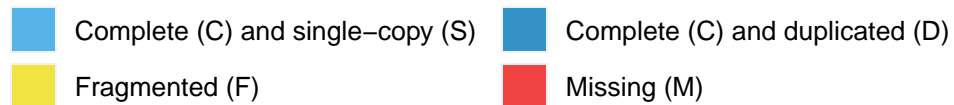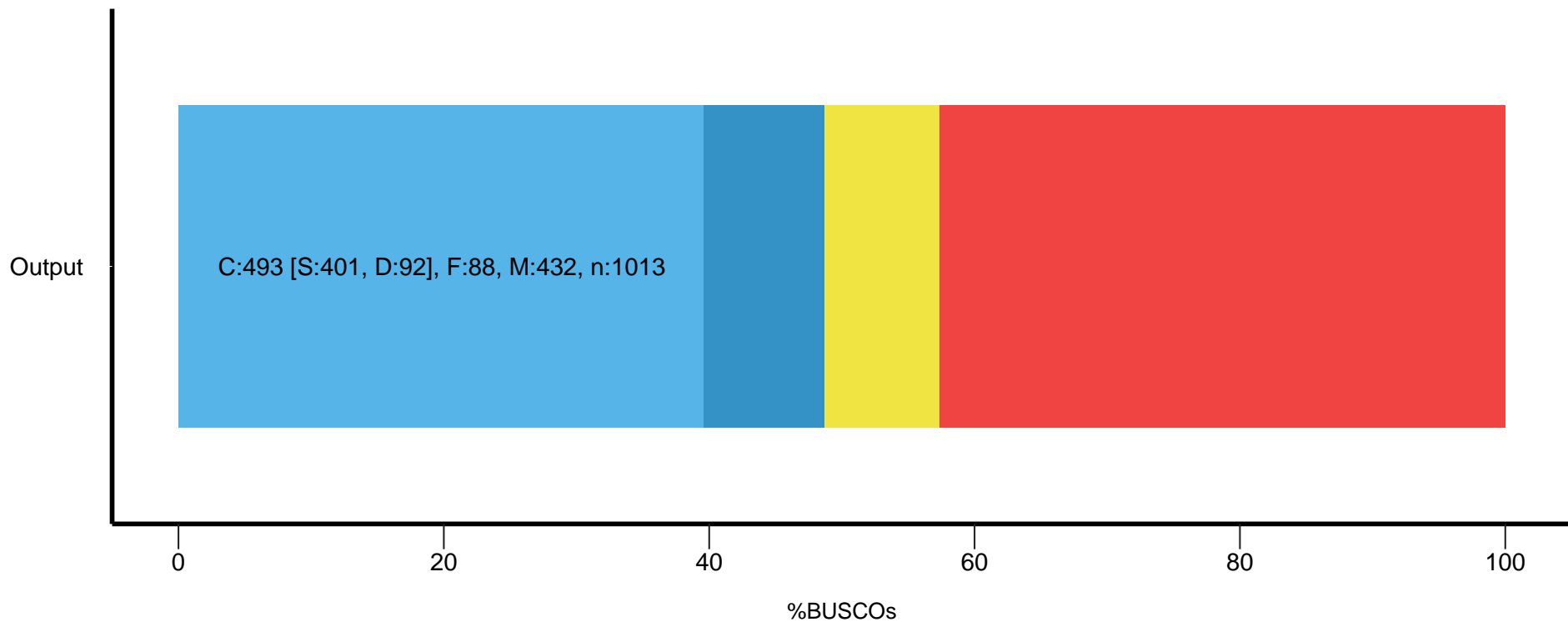

Supplement: S1 Fig — (PDF) [file pone.0279992.s012.pdf]

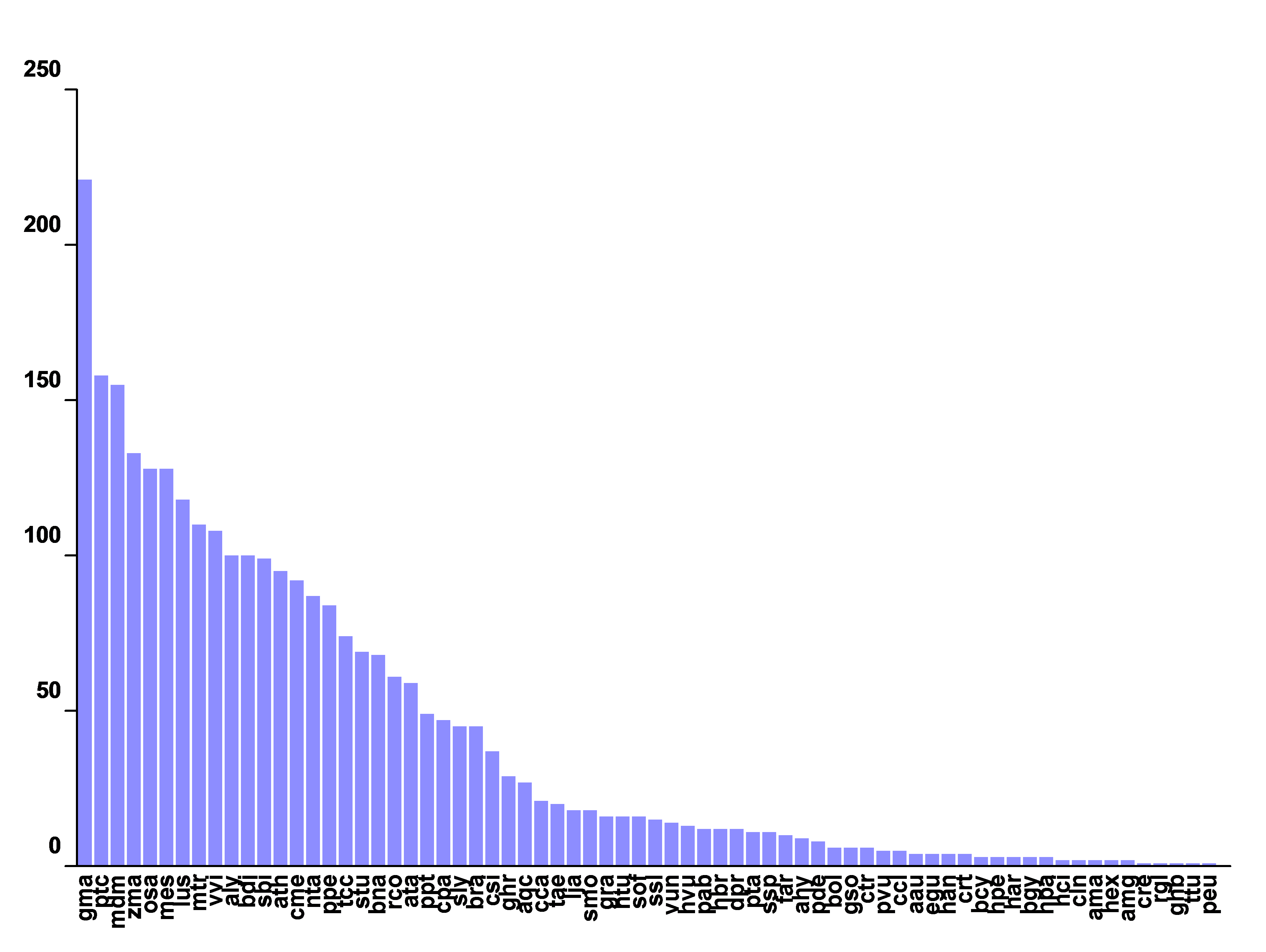

Supplement: S5 Fig — (TIF) [file pone.0279992.s016.tif]
