# Supplementary material for: A tissue-specific profile of miRNAs and their targets related to paeoniaflorin and monoterpenoids biosynthesis in Paeonia lactiflora Pall. by transcriptome, small RNAs and degradome sequencing
Source: PLoS One. 2023 Jan 26;18(1):e0279992. doi: 10.1371/journal.pone.0279992 (PMC9879538; doi:10.1371/journal.pone.0279992)
Supplement: S2 Fig — (DOCX) [file pone.0279992.s013.docx]

A


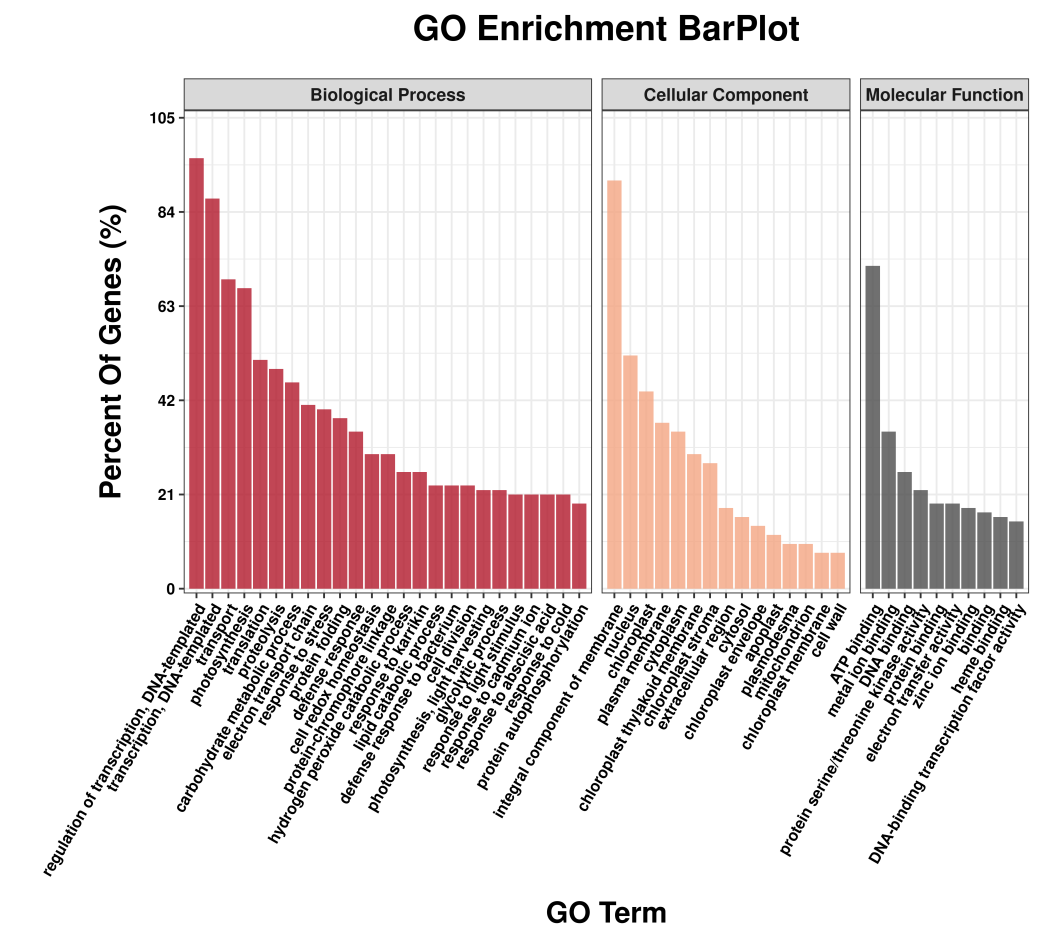


B


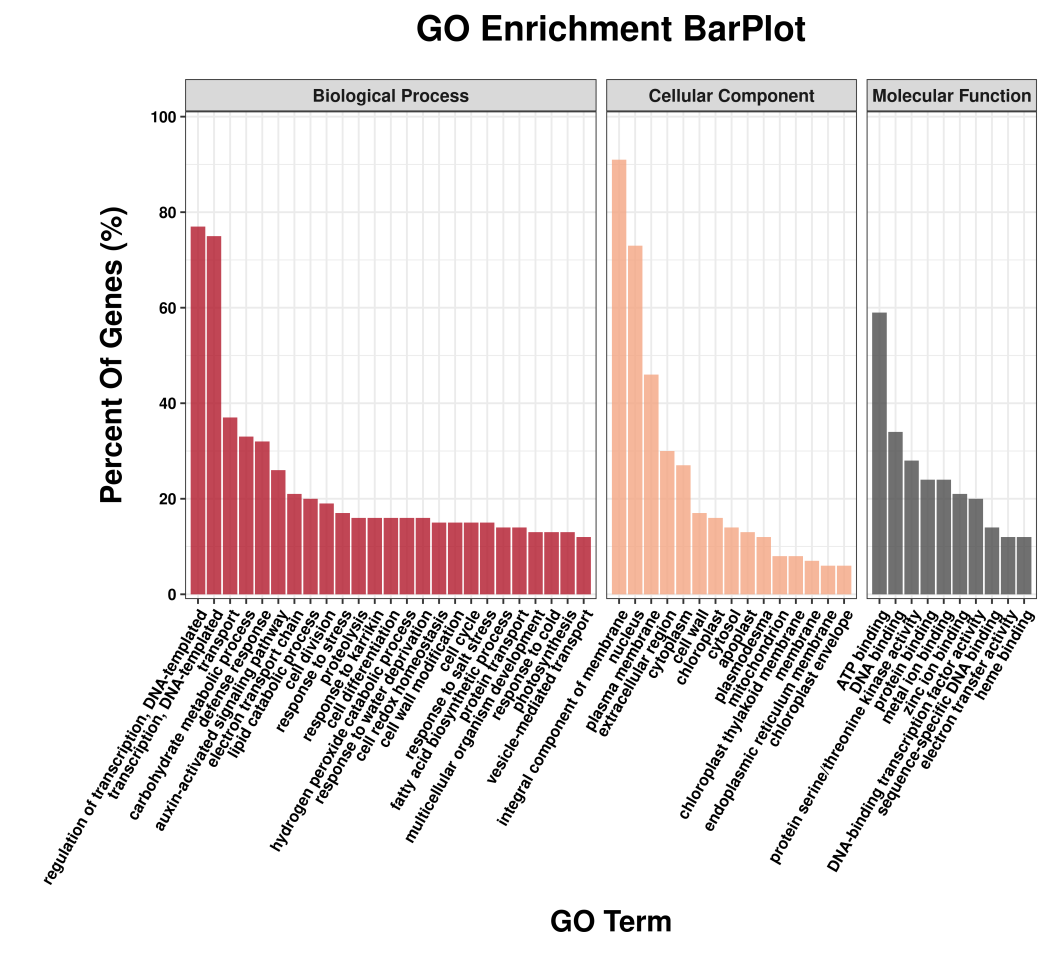


C


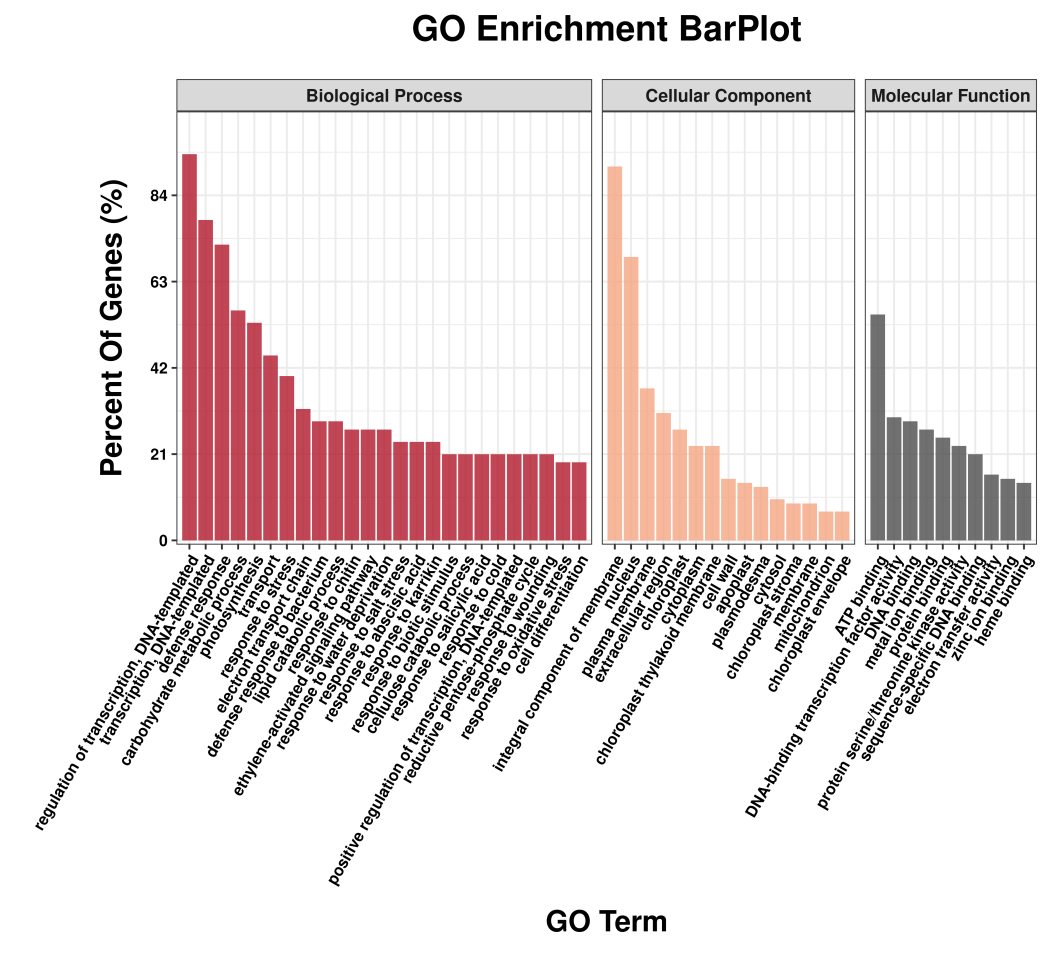


S2 Fig GO enrichment analysis of DEGs between different tissues (A: Leaf vs Root, B Flower vs Root, C Fruit vs Root)
