# Supplementary material for: A tissue-specific profile of miRNAs and their targets related to paeoniaflorin and monoterpenoids biosynthesis in Paeonia lactiflora Pall. by transcriptome, small RNAs and degradome sequencing
Source: PLoS One. 2023 Jan 26;18(1):e0279992. doi: 10.1371/journal.pone.0279992 (PMC9879538; doi:10.1371/journal.pone.0279992)
Supplement: S4 Fig — (PDF) [file pone.0279992.s015.pdf]

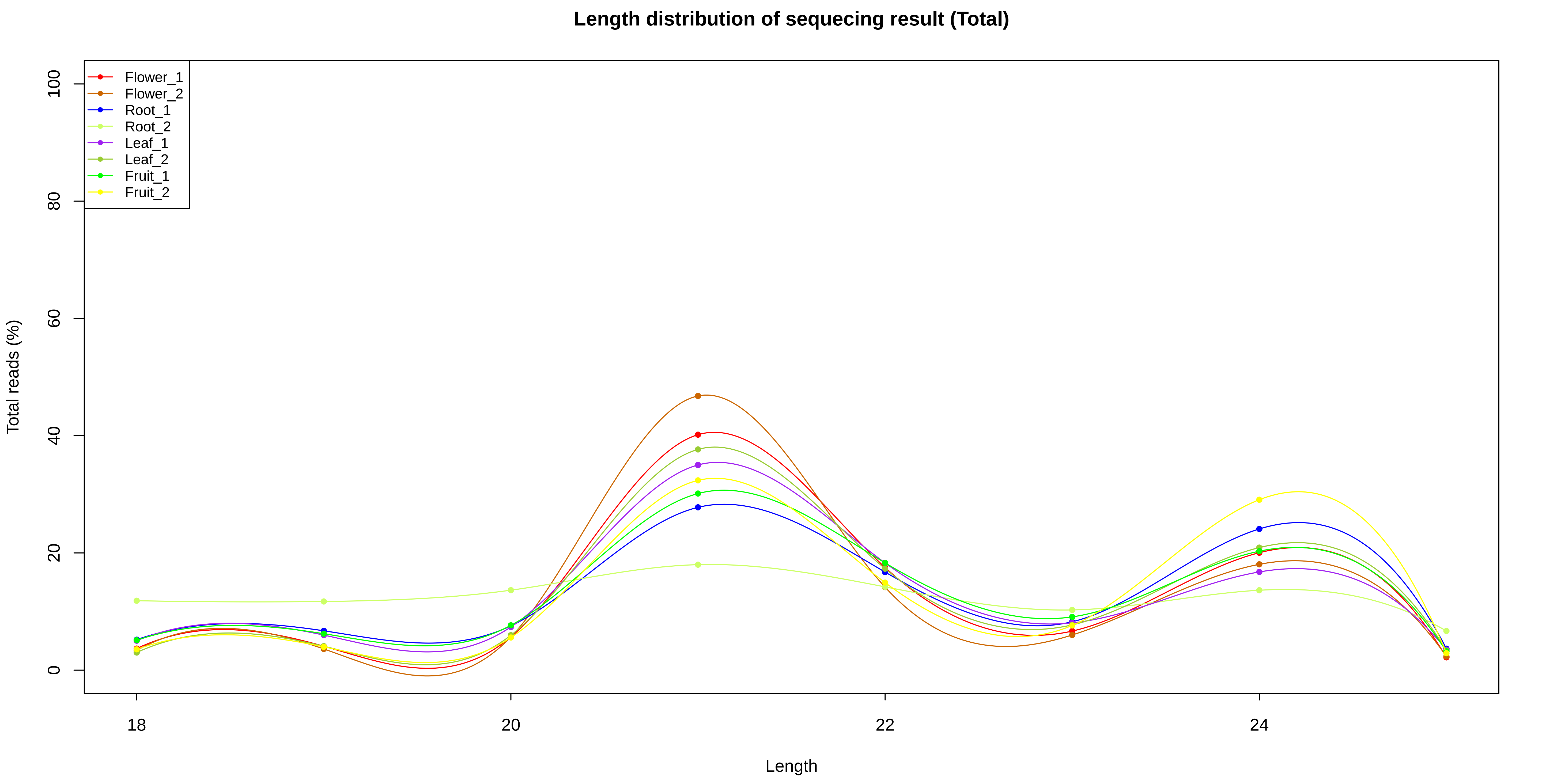
Length distribution of sequecing result (Total)
Flower_1
Flower_2
Root_1
Root_2
Leaf_1
Leaf_2
Fruit_1
Fruit_2
Total reads (%)
0
20
40
60
80
100
18
20
22
24
Length

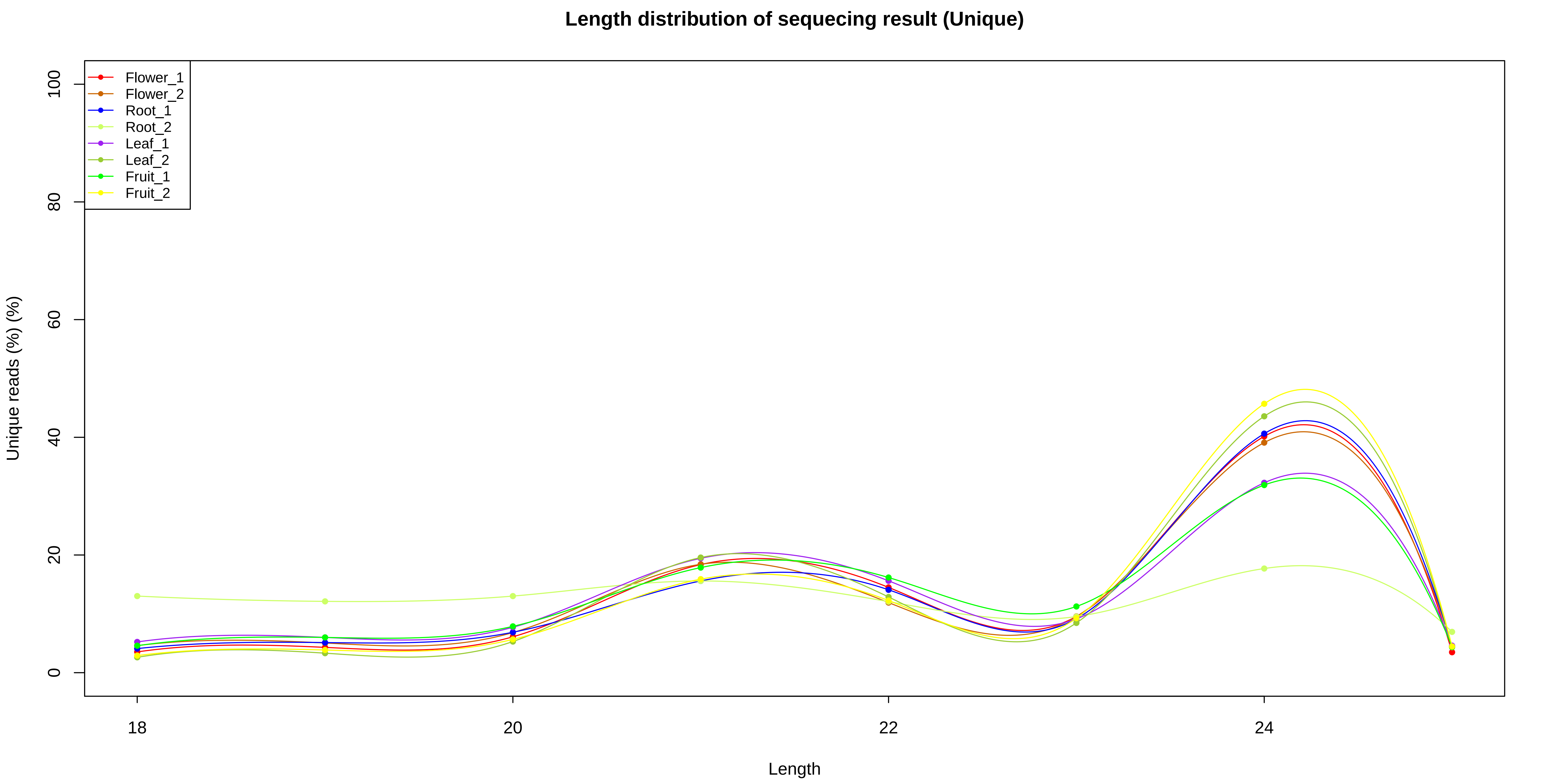
Length distribution of sequecing result (Unique)
Flower_1
Flower_2
Root_1
Root_2
Leaf_1
Leaf_2
Fruit_1
Fruit_2
Unique reads (%) (%)
0
20
40
60
80
100
18
20
22
24
Length
